# The COVID-19 Pandemic and Cancer Patients in Germany: Impact on Treatment, Follow-Up Care and Psychological Burden

**DOI:** 10.3389/fpubh.2021.788598

**Published:** 2022-02-09

**Authors:** Rachel D. Eckford, Andrea Gaisser, Volker Arndt, Michael Baumann, Evelyn Kludt, Katja Mehlis, Jasper Ubels, Eva C. Winkler, Susanne Weg-Remers, Michael Schlander

**Affiliations:** ^1^Division of Health Economics, German Cancer Research Center (DKFZ), Heidelberg, Germany; ^2^German Cancer Information Service, German Cancer Research Center (DKFZ), Heidelberg, Germany; ^3^Unit of Cancer Survivorship, Division of Clinical Epidemiology and Aging Research, German Cancer Research Center (DKFZ), Heidelberg, Germany; ^4^Epidemiological Cancer Registry Baden-Württemberg, German Cancer Research Center (DKFZ), Heidelberg, Germany; ^5^German Cancer Research Center (DKFZ), Heidelberg, Germany; ^6^Department of Medical Oncology, Section of Translational Medical Ethics, National Center for Tumor Diseases, University Hospital Heidelberg, Heidelberg, Germany; ^7^Medical Faculty Mannheim, University of Heidelberg, Mannheim, Germany; ^8^Alfred Weber Institute, Faculty of Economics and Social Sciences, University of Heidelberg, Heidelberg, Germany

**Keywords:** COVID-19, cancer care, changes in treatment, anxiety, depression, health care management

## Abstract

In response to the ongoing coronavirus disease 2019 (COVID-19) pandemic, governments imposed various measures to decrease the rate of disease spread, and health care policy makers prioritized resource allocation to accommodate COVID-19 patients. We conducted a cross-sectional online survey in Germany (July 2020–June 2021) to assess the frequency of changes to cancer care among cancer patients and to explore the psychological impact of the pandemic writ large. Cancer patients who contacted the Cancer Information Service (Krebsinformationsdienst, KID) of the German Cancer Research Center (Deutsches Krebsforschungszentrum, DKFZ) via email were invited to complete an online questionnaire, capturing demographics, cancer specifics (e.g., type, disease phase, primary place of treatment, etc.), and any changes to their medical, follow-up, psycho-oncological or nursing care. General level of psychological distress was measured using the Hospital Anxiety and Depression Scale (HADS) along with face-validated items regarding worries and social isolation specific to the pandemic. In total, 13% of 621 patients reported a change to their treatment or care plan. Of those patients with changes, the majority of changes were made to follow-up care after treatment (56%), to monitoring during treatment (29%) and to psychological counseling (20%). Of the overall sample, more than half of patients (55%) reported symptoms of anxiety and 39% reported symptoms of depression. Patients with a change in cancer care were more likely to report symptoms of depression than those with no change (AOR: 2.18; 95% CI: 1.26–3.76). Concern about the pandemic affecting the quality of health care was a predictor of both anxiety (AOR: 2.76; 95% CI: 1.75–4.35) and depression (AOR: 2.15; 95% CI: 1.43–3.23). Results showed that the majority of cancer patients in our study did not experience a change in their cancer care. However, the level of anxiety and psycho-social burden of cancer patients during the pandemic was high throughout the study period. Our findings underscore the need for health care services and policy makers to assess and to attend cancer patients' medical needs, with added emphasis on patients' psychological and social well-being. This applies particularly in situations where the healthcare system is strained and prioritization is necessary.

## Introduction

The coronavirus disease 2019 (COVID-19) pandemic has had a global impact on health care. The pandemic reached Germany in January 2020 and within 100 days, the number of confirmed cases exceeded 150,000, with over 6,200 deaths (1). The rise in incidence occurred despite unprecedented measures that were taken by both national and regional governments in Germany to control the pandemic. By late March, 2020, business closures were mandated, school classes were relegated to being conducted online, and gatherings of people were greatly restricted. Some of these restrictions were eased by late April; however, a second wave (i.e., a substantial increase in COVID-19 infections) in the fall of 2020 and a third wave in the spring of 2021 kept varying restrictions in place on businesses, schools and social gatherings. By September 23, 2021, over four million cases and more than 93,000 deaths had been confirmed in the country (2).

Within the domain of health care, an attempt to mitigate the potential overload on the healthcare system, particularly in hospitals and intensive care units (ICUs), additional staff were recruited, elective procedures (operations and other medical interventions) were postponed and hospital and ICU capacity was kept available for patients severely sickened with COVID-19 (3). The prioritization of medical resources for COVID-19 patients means potential shortcomings in the care of other vulnerable patient groups, such as cancer patients. Discussion is ongoing over the ethics of resource allocation (4–7). Evidence has shown that delays in cancer treatment can have detrimental health effects. For instance, Hanna et al. found that even a 4 week delay in surgical, systemic or radiation treatment is associated with greater risk of death for seven cancer types (8). Yet, cancer patients may be at greater risk of a COVID-19 infection or related death (9, 10), thus there is a trade-off between patients receiving care and being protected from the risk of infection.

Beyond cancer care, social distancing, regardless of it being government- or self-imposed, potentially has negative mental health consequences (11, 12). Cancer patients are particularly vulnerable irrespective of the COVID-19 pandemic in terms of depression and anxiety (13–15) and also social isolation (16). The added strain of the pandemic, whether it be restricted access to care, fear of being infected with COVID-19, missing contact with other people, among other factors (e.g., financial distress) may compound these already existing issues.

During a pandemic, in the domain of public health and health services research, it is important to gather real-life data from vulnerable groups, such as cancer patients, who might be affected. The present study assessed the frequency of changes to treatment and follow-up care among cancer patients and explored the psychological impact of such changes as well as the psychological impact of the pandemic in general. In addition, we sought to identify possible vulnerable subpopulations to help healthcare professionals and policy makers assess needs and prioritize services to allocate equitable care.

## Materials and Methods

### Design and Study Population

Data was gathered using an anonymized online questionnaire. To obtain estimates with high precision, a sample size of 600 evaluable cases was estimated to be appropriate based on the Clopper–Pearson interval method. Study participants were cancer patients recruited consecutively after they sent an email inquiry regarding their illness to the Cancer Information Service (Krebsinformationsdienst, KID) of the German Cancer Research Center (Deutsches Krebsforschungszentrum, DKFZ). In their email response to these inquiries, staff members of the KID included an invitation to the study with a link to the questionnaire. Inclusion criteria were a confirmed cancer diagnosis, permanent residence in Germany, and age 18 or older. Patients were excluded (i.e., were not asked to participate in the study) if they were undergoing initial diagnostic procedures for suspected cancer, if the study recruiter had doubts about a cancer diagnosis or the potential responder's German language proficiency. Participation in the study was voluntary and could be terminated at any time while taking the questionnaire. All email inquiries were deleted after they had been addressed; therefore, no personal information (names or email addresses) was retained. Furthermore, the link to the questionnaire provided in the invitation email was not personalized, rendering it impossible to know which email recipients participated in the study. Data was collected from July 10, 2020 to June 30, 2021. The principles of the Helsinki Declaration were followed. The ethics committee of the University of Heidelberg approved the study (S-350/2020). In addition, prior to the study being launched, the DKFZ data protection officer reviewed the participant information, consent, and online questionnaire to ensure participant anonymity.

### Online Questionnaire

The questionnaire was programed using the open-sourced web survey application, LimeSurvey, and consisted of five sections: (1) demographics; (2) cancer status (3) experiences with health care during the pandemic (i.e., changes in treatment); (4) psycho-social distress and quality of life; and (5) the financial effects of cancer during the pandemic.

Cancer status items included cancer diagnosis, specific information regarding the cancer (e.g., type, phase, metastasis) and the type of treatment (e.g., which treatment regimen was ongoing or next planned, place of primary treatment). Health care during the pandemic consisted of items about whether the respondent had been infected with COVID-19 or if a family member or friend was infected; whether there had been a change in the treatment or follow-up plan during the pandemic, and, if applicable, what type of change or changes had occurred, as well as specific information about these changes. Types of possible changes to care included: operation, systemic therapy, radiotherapy, progress monitoring during treatment, follow-up after treatment, psycho-social or psycho-oncological counseling, and nursing care. Multiple changes to planned treatment were possible. Questions on the cancer disease (e.g., tumor type, phase of treatment) were informed by evaluations of the routine KID inquiry documentation (17).

Depression and anxiety were assessed using the Hospital Anxiety and Depression Scale (HADS) (18). The German version of the HADS has been validated (19, 20). This measure uses 14-items (seven questions each for the two subscales) with a 4-point Likert scale. Items are scored from 0 to 3 with higher scores indicating higher symptom burden. Scores of 11 or higher per subscale are considered exceeding criteria (i.e., caseness) and scores between eight and ten as being borderline. Authorization to use the HADS scale was obtained. Finally, questions regarding subjective worry as well as social isolation specific to the pandemic were designed *ad hoc* for this study. These questions were face-validated and cognitively pre-tested for comprehensibility prior to the study's launch. The subjective worry items use a 5-point Likert scale (strongly agree to strongly disagree) and items include “As a consequence of the pandemic, I am worried about the possible effects on the quality of my medical care,” “…I am worried that I could be sickened or die from a COVID-19 infection,” and “…I am worried that family or friends could be sickened or die from a COVID-19 infection.” Finally, social isolation items include “Due to restrictions, to what extent do you miss personal contact with relatives, friends, work colleagues, and neighbors?”, “…with other patients and support groups?”, “…with physicians and nurses?”, “…with caregivers, therapists and other helpers?”, “…with personal contacts in the public (e.g., going to a pub, park, concert, theater, shopping?” with a 5-point Likert scale (miss it extremely to do not miss it at all).

### Data Analysis

Statistical analyses were conducted using the statistical program IBM SPSS Statistics (version 27). Descriptive statistics displaying sample sizes and percentages were performed to summarize the responses to demographic and clinical features. Chi-square or Fisher's exact test were used for categorical variables to test associations. Results of these analyses are provided in the Supplementary Material. Correction for confounders was made by multivariate logistic regression analysis. Chi-square significance evaluated at the 0.1 alpha level was included in the regression models to reduce the likelihood of missing potentially associated variables (21). A two-tailed *p*-value < 0.05 was considered statistically significant for the regression analysis. Unadjusted (univariate) and adjusted (multivariate) results are presented.

To examine participants subjective worries during the pandemic, we dichotomized the following responses (agree and strongly agree vs. neutral to strongly disagree) for the questions, “I am worried that I could be sickened or die from the coronavirus infection,” “I am worried that family or friends could be sickened or die from the coronavirus infection,” and “I am worried that the effects of the pandemic could affect the quality of medical treatment.” To consider how COVID-19 restrictions affected patients, we dichotomized the responses (miss it extremely and miss it quite a bit vs. neutral, do not miss it a lot, do not miss it at all) for the question, “How do you feel about the limitations of personal encounters due to the Coronavirus pandemic? Personal contacts with relatives, friends, work colleagues, neighbors…” For the HADS we used the cut-off that included borderline scores (subscale sum scores ≥8) to better capture vulnerable sub-populations, which is consistent with most other publications measuring depression and anxiety during the COVID-19 pandemic (22). Cronbach's alpha was used to demonstrate internal consistency of the HADS.

Analyses presented here focus on whether there was a change in cancer care, on identifying any subgroups vulnerable to changes in care, on the psychological impact of changes to care (patients with changes compared to those without) and on the psychological impact of the pandemic to all patients regardless if there was a change in care, and which sub-groups may be associated with higher rates of psycho-social distress.

## Results

### Patients' Sociodemographic Characteristics and Clinical Features

**Figure 1:**
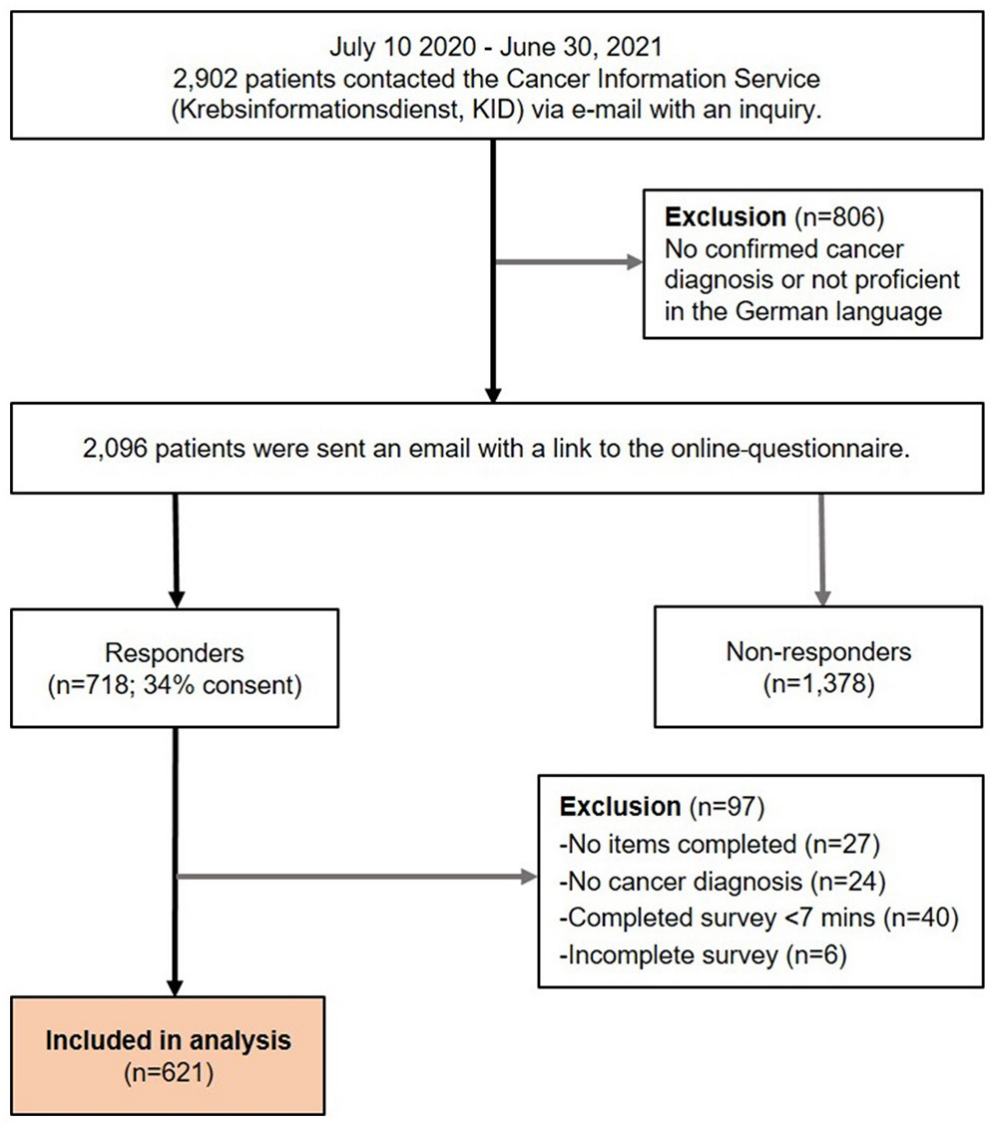
Flow diagram of inclusion of study participants.

In total, 718 patients took the survey, rendering a consent rate of 34%. After data cleaning, 621 patients were included in the analyses. Figure 1 displays a flowchart of participant inclusion.

The demographic characteristics and clinical features of respondents are provided in Table 1. The mean age was 60 years with a standard deviation of 11.8. As this is a convenience sample of cancer patients actively seeking information, the characteristics are neither representative of the German population nor of the cancer patients in Germany with regard to distribution of sex (76% female), age (63% were 41–65 years vs. 36% based on the population in 2019) (23), education (64% university entrance qualification vs. 35% of the population older than 20 years of age) (23) and cancer site (50% breast cancer vs. 20% based on 5-year-prevalence 2017) (24). Regarding health insurance, 17.6% had private health insurance vs. 11% based on the population (25).

**Table 1:** Patient demographic characteristics and clinical features.

**Total sample (*****N*** **=** **621)**		***M***	***SD***
		59.5	11.8
		***n***	**%**
Age (*n* = 595)	18–40	39	6.6%
	41–65	373	62.7%
	66+	183	30.8%
Gender (*n* = 619)	Male	147	23.7%
	Female	472	76.3%
Education (*n* = 610)	Secondary general school-leaving certificate	62	10.2%
	Intermediate school-leaving certificate	159	26.1%
	University entrance qualification	389	63.8%
Living situation (*n* = 603)	Lives alone	111	18.4%
	Lives with another/others	492	81.6%
		***M***	***SD***
		0.26	0.63
		***n***	**%**
Minor-aged kids living at home (*n* = 621)	No	514	82.8%
	Yes	107	17.2%
Employment (*n* = 610)	Employed	271	44.4%
	Self-employed	41	6.7%
	Retired	239	39.2%
	Unemployed	59	9.7%
Health insurance (*n* = 615)	Private	108	17.6%
	Statutory	377	61.3%
	Statutory with private supplemental	106	17.2%
	Co-insured free-of-charge	24	3.9%
Type of cancer (*n* = 619)	Breast cancer	310	50.1%
	Prostate cancer	66	10.7%
	Colon cancer	23	3.7%
	Lung cancer	19	3.1%
	Other	201	32.5%
Metastatic cancer (*n* = 615)	No	414	67.3
	Yes or suspected	157	25.5
	Do not know	44	7.2
Cancer phase (*n* = 612)	After diagnosis or during initial treatment	197	32.2%
	Initial treatment completed	245	40.0%
	Relapse/relapse treatment	85	13.9%
	Advanced disease/palliative treatment	68	11.1%
	Do not know	17	2.8%
Setting for main treatment (*n* = 617)	Hospital inpatient	31	5.0%
	Hospital outpatient	187	30.3%
	Oncology practice	160	25.9%
	Other	239	38.7%
Observation period (*n* = 621)	Before second wave (Jul. 10–Nov. 1, 2020)	185	29.8%
	Second wave (Nov. 2, 2020–Mar. 11, 2021)	283	45.6%
	Third wave (Mar. 12, 2021–Jun. 30, 2021)	153	24.6%
Sickened by COVID-19 (*n* = 603)	No or do not know	596	98.8%
	Yes	7	1.2%
Family, friend or acquaintance sickened by COVID-19 (*n* = 621)	No or do not know Yes	482 139	77.6% 22.4%

One or multiple cancer treatments or examinations were ongoing or planned for participants. Treatments included surgery to remove primary tumor or metastases (100, 16%); systemic therapy before planned surgery (52, 8.4%), after planned surgery (207, 33.3%), and for advanced disease (112, 18.0%); and radiotherapy to tumor region or to metastases (102, 16.4%). Examinations included monitoring during therapy (138, 22.2%), follow-up in aftercare (247, 39.8%), or patients might be in a phase of “wait and see” (50, 8.1%). Uncertainty of next planned treatment was reported by 18 patients (2.9%). Regarding the setting for primary treatment, 30.3% of participants were treated in hospital outpatient clinics, 25.9% in an oncology practice, and 5% as hospital inpatient. The plurality of participants reported “other” as setting (38.7%). Upon examination of what this comprised—information was provided in free-text on the online questionnaire—other settings consisted of specialized clinics corresponding to cancer type (e.g., breast cancer patients being treated in gynecology clinics, prostate cancer patients in urology).

### Changes to Cancer Care

Overall, 79 respondents (12.9%) reported a change (i.e., a postponement, cancellation, or another type of treatment/mode of communication, such as videoconferencing) to a scheduled treatment, examination or care plan. The changes were as follows: 10 patients reported a change to a planned operation (1.6% of sample; 12.7% of those reporting a change); 14 reported a change to systemic therapy (2.3%; 17.7%); one person reported a change to radiotherapy (0.2%; 1.3%); 23 had a change to progress monitoring during treatment (3.7%; 29.1%) and 44 to a follow-up after treatment (7.1%; 55.7%); 16 patients reported a change to a psycho-social or psycho-oncological counseling appointment (2.6%: 20.3%); and three patients reported a change to care by nursing service (0.5%; 3.8%). Twenty-four patients (38.8%) of those reporting a change in treatment reported more than one change. Table 2 displays results of change in cancer treatment.

**Table 2:** Change in care or to follow-up.

**Total sample (*****N*** **=** **621)**		***n***	**% Entire sample**	**% Patients with change**
Change to care during pandemic (*n* = 611)	No	532	87.1%	
	Yes	79	12.9%	
No. of changes (*n* = 78)	One	54	8.7%	69.2%
	Two or more	24	3.9%	30.8%
Type of tx change^*^	Operation	10	1.6%	12.7%
	Systemic therapy	14	2.3%	17.7%
	Radiotherapy	1	0.2%	1.3%
	Progress monitoring during treatment	23	3.7%	29.1%
	Follow-up after treatment	44	7.1%	55.7%
	Psycho-social or psycho-oncological counseling	16	2.6%	20.3%
	Nursing care	3	0.5%	3.8%

* *Multiple changes are possible*.

In regard to which socio-demographic and clinical features were associated with having a change in treatment, only the phase of treatment was statistically significant. Patients in the phase of treatment after diagnosis or during initial treatment were the group with the lowest percentage of changes in care (6.2%) and those reporting to be in the phase where initial treatment had been completed (e.g., follow-up care) had the highest (18.9%). Those in the relapse phase and those with advanced disease were in the middle (15.2 and 11.9% with changes, respectively). The odds of a patient having a change in care was 3.50 times greater in the phase after initial treatment was completed (95% CI 1.80–6.82, *p* < 0.001) and 2.68 times greater in the advanced disease phase (95% CI 1.10–6.53, *p* = 0.030), compared to those in the initial phase of treatment. The results are consistent with the finding above that most changes were to follow-up examinations. No other statistically significant associations were found among the sociodemographic and clinical factors (e.g., not age, form of health insurance, type of cancer, etc.) regarding a change in cancer care.

### Subjective Distress and Missing Contact With Others

**Figure 2:**
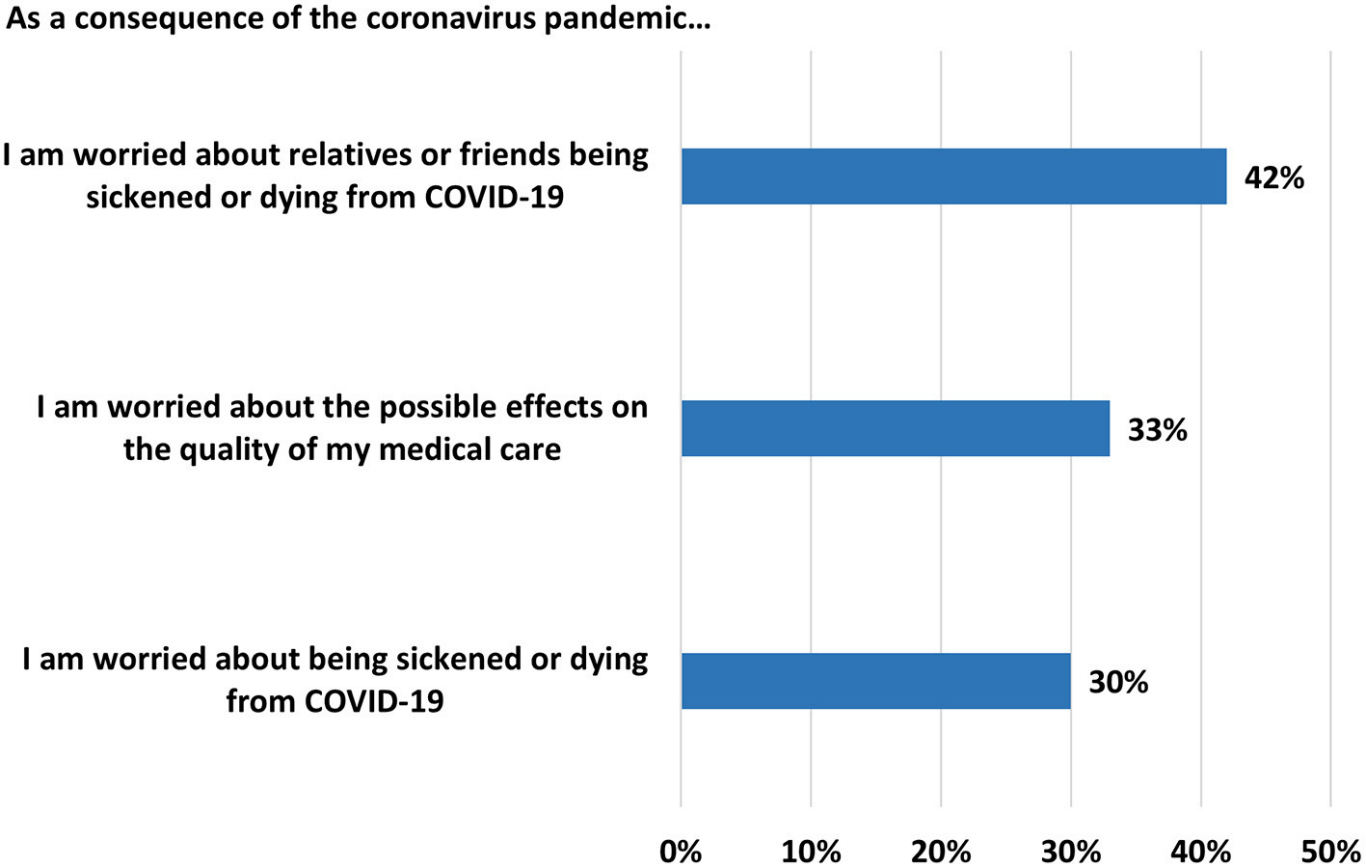
Percent of agreement or strong agreement to subjective concerns during the pandemic.

In total, 30% of participants reported concern about being sickened or dying from a COVID-19 infection; a greater percentage (42%) reported worry that family or friends could be infected or die. One third (33%) were worried that as a consequence of the corona pandemic, the quality of their medical care would be impacted (see Figure 2). In terms of group comparisons, age, gender, type of cancer and observation period were predictor variables for concern of a COVID-19 infection. Gender, whether or not the patient had metastatic cancer, treatment phase, observation phase and whether a family member or friend had a confirmed COVID-19 diagnosis were covariates for worry concerning family and friends. However, after adjustment, gender was found to be a predictor for concern for others (AOR: 0.56; 95% CI: 0.36–0.86; *p* = 0.008), but not for concern for oneself. The odds of being concerned about a COVID-19 infection were less during the third observation period (i.e., during the third wave, but when vaccines were available; AOR: 0.54; 95% CI: 0.31–0.92; *p* = 0.023), and the odds of being concerned for others were higher during the second observation period (i.e., during the second wave; AOR: 1.82; 95% CI: 1.22–2.74; *p* = 0.004). As for worry that the pandemic would affect the quality of patients' medical treatment, age, having minor-aged children living at home, and type of health insurance were retained for multivariate analysis. However, after adjustment, only type of health insurance was statistically significant. Patients with statutory insurance were more likely to report concern regarding the quality of their medical treatment (AOR: 2.35; 95% CI: 1.37–4.04; *p* = 0.002). Although not statistically significant, patients in the 18–40 age group were more than twice as likely to report concern regarding the quality of care than those in the age group 66 and older (AOR: 2.18; 95% CI: 1.00–4.77); *p* = 0.052. Tables 3–5 show results of these logistic regression analyses.

**Table 3:** Logistic regression of associated factors for patient worry regarding being sickened or dying from COVID-19^a^.

				**Unadjusted results**		**Adjusted results**	
**Variables**	**Category**	**Total, *N***	**Worry COVID-19 (Self)^**b**^ *n* (%)**	**Odds ratio** **95% CI**	***P*-value**	**Odds ratio** **95% CI**	***P*-value**
Age	18–40	39	18 (46.2)	–	–	–	–
	41–65	372	110 (29.6)	0.49 (0.25–0.96)	0.036	0.58 (0.29–1.16)	0.121
	66+	181	50 (27.6)	0.44 (0.22–0.90)	0.025	0.66 (0.31–1.42)	0.292
Gender	Male	145	33 (22.8)	0.62 (0.40–0.96)	0.033	0.74 (0.39–1.40)	0.351
	Female	471	151 (32.1)	–	–	–	–
Type of cancer	Breast cancer	310	102 (32.9)	–	–	–	–
	Prostate cancer	65	10 (15.4)	0.37 (0.18–0.76)	0.006	0.40 (0.14–1.08)	0.072
	Colon cancer	22	8 (36.4)	1.16 (0.47–2.87)	0.739	1.50 (0.56–4.01)	0.421
	Lung cancer	19	3 (15.8)	0.38 (0.11–1.34)	0.133	0.27 (0.06–1.21)	0.086
	Other	200	61 (30.5)	0.90 (0.61–1.31)	0.570	0.98 (0.63–1.53)	0.939
Observation period	Before second wave (Jul. 10–Nov. 1, 2020)	184	56 (30.4)	–	–	–	–
	Second wave (Nov. 2, 2020–Mar. 11, 2021)	281	100 (35.6)	1.26 (0.85–1.88)	0.250	1.38 (0.90–2.10)	0.139
	Third wave (Mar. 12, 2021–Jun. 30, 2021)	153	29 (19.0)	0.54 (0.32–0.89)	0.016	0.54 (0.31–0.92)	**0.023**

a *I am worried that I could be sickened or die from the coronavirus infection*.

b *Agree to strongly agree. Bold value indicates p < 0.05*.

**Table 4:** Logistic regression of associated factors for patient worry regarding relatives and friends being sickened or dying from COVID-19^a^.

				**Unadjusted results**		**Adjusted results**	
**Variables**	**Category**	**Total, *N***	**Worry COVID-19 (Others)^**b**^ *n* (%)**	**Odds ratio** **95% CI**	***P*-value**	**Odds ratio** **95% CI**	***P*-value**
Gender	Male	144	46 (31.9)	0.57 (0.39–0.85)	0.006	0.56 (0.36–0.86)	**0.008**
	Female	471	212 (45.0)	–	–	–	–
Metastatic cancer	No	411	186 (45.3)	–	–	–	–
	Yes or suspected	156	57 (36.5)	0.70 (0.48–1.02)	0.062	0.70 (0.43–1.15)	0.157
	Do not know	44	14 (31.8)	0.56 (0.29–1.10)	0.091	0.57 (0.28–1.18)	0.132
Phase of treatment	After diagnosis or during initial treatment	196	70 (35.7)	–	–	–	–
	Initial treatment completed	243	118 (48.6)	1.70 (1.16–2.50)	0.007	1.58 (1.06–2.36)	**0.026**
	Relapse/relapse treatment	85	36 (42.2)	1.32 (0.79–2.22)	0.292	1.64 (0.92–2.91)	0.092
	Advanced disease/palliative treatment	67	27 (40.3)	1.22 (0.69–2.15)	0.502	1.53 (0.77–3.02)	0.222
Observation period	Before second wave (Jul. 10–Nov. 1, 2020)	184	69 (37.5)	–	–	–	–
	Second wave (Nov. 2, 2020–Mar. 11, 2021)	281	141 (50.2)	1.68 (1.15–2.45)	0.007	1.82 (1.22–2.74)	**0.004**
	Third wave (Mar. 12, 2021–Jun. 30, 2021)	152	50 (32.9)	0.82 (0.52–1.28)	0.380	0.80 (0.50–1.29)	0.354
Family, friend or acquaintance sickened by COVID-19	No or do not know	478	190 (39.7)	–	–	–	–
	Yes	139	70 (50.4)	1.54 (1.05–2.25)	0.026	1.34 (0.89–2.02)	0.162

a *I am worried that family or friends could be sickened or die from the coronavirus infection*.

b *Agree to strongly agree. Bold value indicates p < 0.05*.

**Table 5:** Logistic regression of associated factors for patient worry regarding effects of the pandemic affecting the quality of medical treatment^a^.

				**Unadjusted results**		**Adjusted results**	
**Variables**	**Category**	**Total, *N***	**Worry quality of care^**b**^ *n* (%)**	**Odds ratio** **95% CI**	***P*-value**	**Odds ratio** **95% CI**	***P*-value**
Age	18–40	39	20 (51.3)	–	–	–	–
	41–65	372	125 (33.6)	0.48 (0.25–0.93)	0.031	0.54 (0.27–1.10)	0.092
	66+	180	49 (27.2)	0.36 (0.18–0.72)	0.004	0.46 (0.21–1.00)	0.052
Minor-aged kids at home	No	511	159 (31.1)	–	–	–	–
	Yes	106	44 (41.5)	1.57 (1.02–2.41)	0.039	1.28 (0.79–2.08)	0.313
Type of insurance	Private	105	20 (19.0)	–	–	–	–
Health insurance	Statutory	376	143 (38.0)	2.61 (1.54–4.43)	<0.001	2.35 (1.37–4.04)	**0.002**
	Statutory with private as supplement	106	30 (28.3)	1.68 (0.88–3.20)	0.116	1.69 (0.88–3.25)	0.113

a *I am worried that the effects of the pandemic could affect the quality of medical treatment*.

b *Agree to strongly agree. Bold value indicates p < 0.05*.

**Figure 3:**
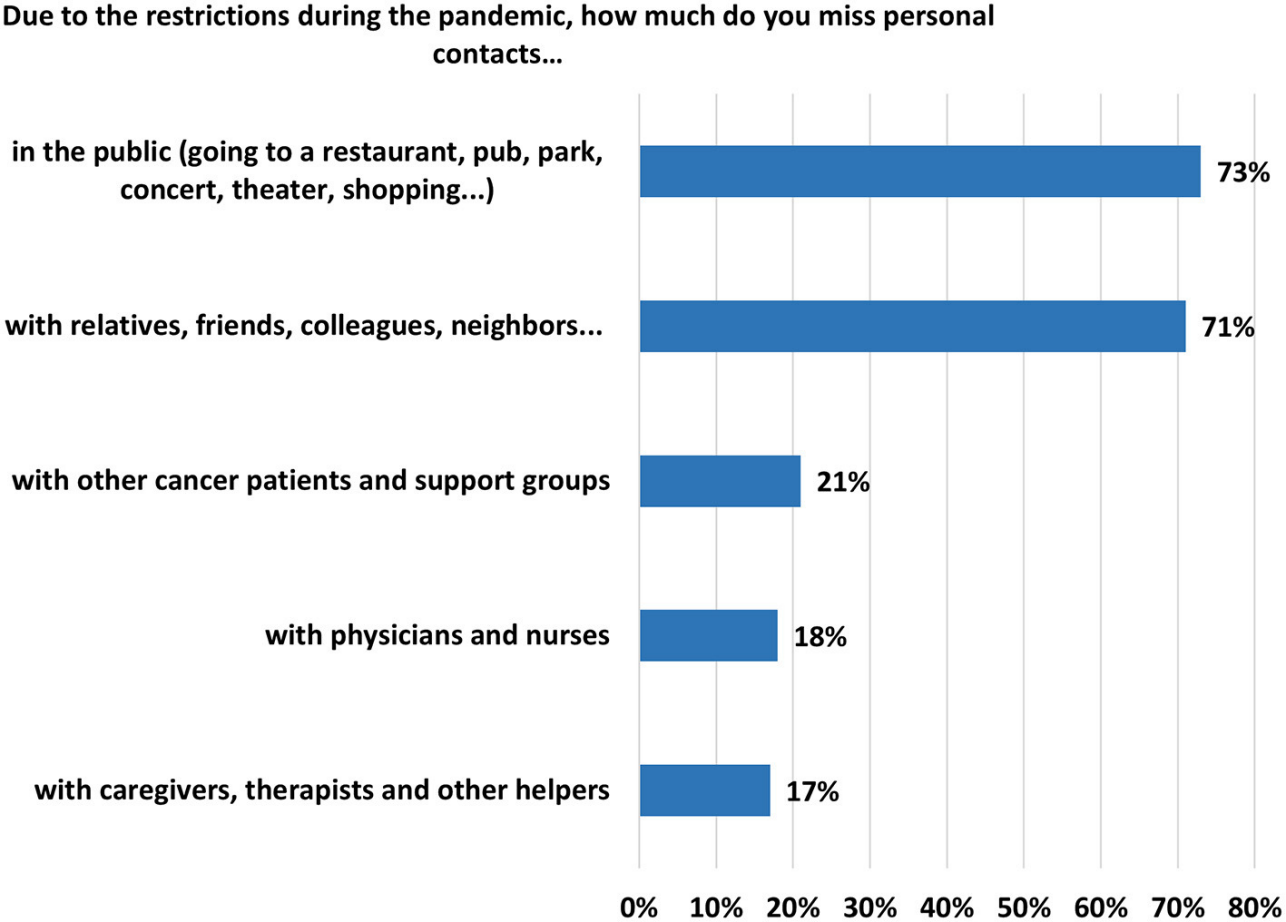
Agreement or strong agreement to missing personal contacts due to restrictions during the pandemic (“miss it quite a bit or very much”).

With regard to how restrictions during the pandemic affected patients, almost three quarters of patients reported they missed public outings and personal contact with family, friends, colleagues, and neighbors (Figure 3). Examining group comparisons for patients missing contact with others outside the home due to COVID restrictions, age, gender, employment status, type of cancer, having a family or friend sickened by COVID-19, and the time point in the pandemic were adjusted. Only gender (AOR: 0.46; 95% CI: 0.24–0.88; *p* = 0.019) and observation period (second wave: AOR 3.20; 95% CI: 2.03–5.04; *p* < 0.001; third wave: AOR: 2.56; 95% CI: 1.53–4.28; *p* < 0.001) were statistically significant. See Table 6 for results of the logistic regression analysis.

**Table 6:** Logistic regression of associated factors for patient missing contacts with relatives, friends, work colleagues, neighbors^a^.

				**Unadjusted results**		**Adjusted results**	
**Variables**	**Category**	**Total, *N***	**Missing Contact^**b**^ *n* (%)**	**Odds ratio** **95% CI**	***P*-value**	**Odds ratio** **95% CI**	***P*-value**
Age	18–40	39	31 (79.5)	–	–	–	–
	41–65	373	272 (72.9)	0.70 (0.31–1.56)	0.379	0.68 (0.29–1.61)	0.381
	66+	182	118 (64.8)	0.48 (0.21–1.10)	0.081	0.83 (0.30–2.32)	0.724
Gender	Male	147	80 (54.4)	0.39 (0.26–0.57)	<0.001	0.46 (0.24–0.88)	**0.019**
	Female	471	356 (75.6)	–	–	–	–
Employment status	Employed	271	204 (75.3)	–	–	–	–
	Self-employed	40	29 (72.5)	0.87 (0.41–1.83)	0.705	0.79 (0.35–1.78)	0.565
	Retired	239	158 (66.1)	0.64 (0.44–0.94)	0.023	0.71 (0.41–1.26)	0.243
	Unemployed or not employed	59	38 (64.4)	0.59 (0.33–1.08)	0.089	0.68 (0.35–1.36)	0.277
Type of cancer	Breast cancer	310	235 (75.8)	–	–	–	–
	Prostate cancer	66	30 (45.5)	0.27 (0.15–0.46)	<0.001	0.54 (0.23–1.28)	0.159
	Colon cancer	22	16 (72.7)	0.85 (0.32–2.25)	0.745	1.27 (0.42–3.88)	0.671
	Lung cancer	19	16 (84.2)	1.70 (0.48–6.00)	0.408	2.22 (0.59–8.34)	0.236
	Other type	201	140 (69.7)	0.73 (0.49–1.09)	0.125	0.82 (0.50–1.33)	0.414
Observation period	Before second wave (Jul. 10–Nov. 1, 2020)	185	105 (56.8)	–	–	–	–
	Second wave (Nov. 2, 2020–Mar. 11, 2021)	282	217 (77.0)	2.54 (1.70–3.80)	<0.001	3.20 (2.03–5.04)	**<0.001**
	Third wave (Mar. 12, 2021–Jun. 30, 2021)	153	116 (75.8)	2.39 (1.49–3.82)	<0.001	2.56 (1.53–4.28)	**<0.001**
Family, friend or	No or do not know	481	327 (68.0)	–	–	–	–
acquaintance sickened	Yes	139	111 (79.9)	1.87 (1.18–2.95)	0.007	1.59 (0.97–2.60)	0.065
by COVID-19							

a *How do you feel about the limitations of personal encounters due to the Corona pandemic? Personal contacts with relatives, friends, work colleagues, neighbors*.

b *Miss it extremely to miss it quite a bit. Bold value indicates p < 0.05*.

### Anxiety and Depression

Internal consistency for the HADS was high for the anxiety and depression items (α = 0.87 and 0.88, respectively). The mean anxiety score was 8.2 (*SD*, 4.4) and the mean depression score was 6.8 (*SD*, 4.5). A total of 339 respondents (54.6%) exceeded criteria for having symptoms of anxiety when including borderline cases (score ≥ 8). For symptoms of depression, 241 participants (38.8%) exceeded criteria.

Table 7 shows group comparisons for those who reported symptoms of anxiety. For the multivariate analysis, age, gender, level of education, having children under age 18 living at home, employment status, type of cancer, whether the cancer was metastatic were adjusted. Other factors included if there was a change in cancer care, subjective worry over being infected with COVID-19 or having family or friends infected, concern for the quality of medical care, and missing contact with others. Results indicated that patients with an intermediate school leaving certificate were more likely to report symptoms of anxiety (AOR: 1.83; 95% CI: 1.17–2.85; *p* = 0.008) compared to those with a higher education (university entrance qualification). In addition, those who reported worrying about the quality of their medical care were more likely to report symptoms of anxiety (AOR: 2.76; 95% CI: 1.75–4.35; *p* < 0.001).

**Table 7:** Logistic regression of associated factors for symptoms of anxiety^a^.

				**Unadjusted results**		**Adjusted results**	
**Variables**	**Category**	**Total, *N***	**Yes^**b**^ *n* (%)**	**Odds ratio** **95% CI**	***P*-value**	**Odds ratio** **95% CI**	***P*-value**
Age	18–40	39	26 (66.7)	–	–	–	–
	41–65	373	218 (58.4)	0.70 (0.35–1.41)	0.322	0.90 (0.40–2.06)	0.813
	66+	183	82 (44.8)	0.41 (0.20–0.84)	0.015	0.89 (0.32–2.46)	0.826
Gender	Male	147	64 (43.5)	0.56 (0.38–0.81)	0.002	0.85 (0.44–1.63)	0.625
	Female	472	274 (58.1)	–	–	–	–
Education	Secondary general school-leaving certificate	62	28 (45.2)	0.76 (0.44–1.31)	0.323	1.05 (0.54–2.03)	0.884
	Intermediate school-leaving certificate	159	103 (64.8)	1.70 (1.16–2.49)	0.006	1.83 (1.17–2.85)	**0.008**
	University entrance qualification	389	202 (51.9)	–	–	–	–
Minor-aged kids at home	No	514	267 (51.9)	–	–	–	–
	Yes	107	72 (67.3)	1.90 (1.23–2.95)	0.004	1.36 (0.80–2.30)	0.263
Employment status	Employed	271	163 (60.1)	–	–	–	–
	Self-employed	41	26 (63.4)	1.15 (0.58–2.27)	0.690	1.29 (0.58–2.83)	0.529
	Retired	239	110 (46.0)	0.56 (0.40–0.80)	0.001	0.67 (0.38–1.17)	0.157
	Unemployed or not employed	59	33 (55.9)	0.84 (0.48–1.48)	0.550	0.84 (0.42–1.69)	0.631
Type of cancer	Breast cancer	310	181 (58.4)	–	–	–	–
	Prostate cancer	66	23 (34.8)	0.38 (0.22–0.66)	0.001	0.70 (0.28–1.72)	0.434
	Colon cancer	23	11 (47.8)	0.65 (0.28–1.53)	0.326	0.54 (0.18–1.59)	0.263
	Lung cancer	19	10 (52.6)	0.79 (0.31–2.00)	0.622	1.10 (0.35–3.44)	0.864
	Other type	201	112 (55.7)	0.90 (0.63–1.28)	0.552	0.96 (0.60–1.53)	0.867
Metastatic cancer (*n* = 615)	No	414	231 (55.8)	–	–	–	–
	Yes or suspected	157	74 (47.1)	0.71 (0.49–1.02)	0.064	0.79 (0.51–1.24)	0.307
	Do not know	44	32 (72.7)	2.11 (1.06–4.22)	0.034	2.08 (0.94–4.61)	0.070
Change in cancer care	No	532	273 (51.3)	–	–	–	–
	Yes	79	61 (77.2)	3.22 (1.85–5.59)	<0.001	1.77 (0.94–3.31)	0.076
Worry about corona infection	No	433	208 (48.0)	–	–	–	–
	Yes	185	129 (69.7)	2.49 (1.73–3.59)	<0.001	1.43 (0.85–2.40)	0.178
Worry about family/friends corona infection	No	357	187 (45.2)	–	–	–	–
	Yes	260	150 (73.9)	2.15 (1.55–3.00)	<0.001	1.24 (0.78–1.99)	0.363
Worry about possible effects on quality of medical care	No	414	167 (46.8)	–	–	–	–
	Yes	203	170 (65.4)	3.44 (2.38–4.96)	<0.001	2.76 (1.75–4.35)	**<0.001**
Missing contact with relatives, friends, etc.	No	182	83 (45.6)	–	–	–	–
	Yes	438	255 (58.2)	1.66 (1.17–2.35)	0.004	1.31 (0.87–2.00)	0.199

a *Hospital Anxiety and Depression Scale—Anxiety*.

b *Anxiety score ≥ 8. Bold value indicates p < 0.05*.

For factors potentially associated with depression, living alone vs. living with another/others and whether the cancer had spread were predictor variables (see Table 8). In addition, having a change in cancer care, worry of a COVID-19 infection for oneself or family or friends, and worry over quality of cancer care were included in the multivariate analysis. Similar to those reporting symptoms of anxiety, worry over the quality of care (AOR: 2.15; 95% CI: 1.43–3.23; *p* < 0.001) was associated with depression. In addition, having a change in cancer care (AOR: 2.18; 95% CI: 1.26–3.76; *p* = 0.005) and worry over family or friends being infected (AOR: 1.76; 95% CI: 1.12–2.74; *p* = 0.013) were associated factors, although being worried about oneself was not. Finally, those who did not know whether the cancer was metastatic were more likely to report symptoms of depression (AOR: 3.06; 95% CI: 1.53–6.12; *p* = 0.002).

**Table 8:** Logistic regression of associated factors for symptoms of depression^a^.

				**Unadjusted results**		**Adjusted results**	
**Variables**	**Category**	**Total, *N***	**Yes^**b**^ *n* (%)**	**Odds ratio** **95% CI**	***P*-value**	**Odds ratio** **95% CI**	***P*-value**
Living situation	Lives alone	111	51 (45.9)	1.45 (0.96–2.19)	0.081	1.42 (0.90–2.22)	0.130
	Lives with others	492	182 (37.0)	–	–	–	–
Metastatic cancer	No	414	152 (36.7)	–	–	–	–
	Yes or suspected	157	59 (37.6)	1.04 (0.71–1.52)	0.848	1.08 (0.71–1.64)	0.714
	Do not know	44	28 (63.6)	3.02 (1.58–5.76)	0.001	3.06 (1.53–6.12)	**0.002**
Change in cancer care	No	532	188 (35.3)	–	–	–	–
	Yes	79	50 (63.3)	3.16 (1.93–5.15)	<0.001	2.18 (1.26–3.76)	**0.005**
Worry about corona infection	No	433	148 (34.2)	–	–	–	–
	Yes	185	91 (49.2)	1.86 (1.31–2.65)	<0.001	1.00 (0.62–1.61)	0.991
Worry about family/friends corona infection	No	357	111 (31.1)	–	–	–	–
	Yes	260	128 (49.2)	2.15 (1.54–2.99)	<0.001	1.76 (1.12–2.74)	**0.013**
Worry about possible effects on quality of medical care	No	414	124 (30.0)	–	–	–	–
	Yes	203	115 (56.7)	3.06 (2.16–4.33)	<0.001	2.15 (1.43–3.23)	**<0.001**

a *Hospital Anxiety and Depression Scale—Depression*.

b *Anxiety score ≥ 8. Bold value indicates p < 0.05*.

## Discussion

This study aimed to investigate how, over the course of a year, the pandemic affected cancer patients in terms of them receiving medical, follow-up care or psycho-oncological counseling. We presented the rates of changes made to care. Specifically, was there a change to care during the pandemic? For which type of cancer care was the change (or changes) made? We also looked at demographic and clinical features to identify possible sub-populations vulnerable to changes in care. Secondly, we examined the status of the study population's mental health, namely anxiety and depression, but also subjective distress unique to the pandemic (e.g., concerns about being infected, about family or friends being infected, or about the effects of the pandemic worsening the quality of cancer care), and missing contact with others. We compared those who had changes to care vs. those who did not. In addition, we explored subgroups vulnerable to psychological burden—regardless of changes to care—by comparing demographic and clinical features, as well as considering the phase of the pandemic.

### Changes to Cancer Care

Overall, 79 respondents (12.9%) reported a change to a treatment, examination or care plan. After comparing demographic and clinical factors, only the phase of treatment was associated with such changes. When comparing those who reported at least one change in planned cancer care with those who did not, the highest proportion of change occurred for patients reporting to be in the phase where initial treatment had been completed (18.9%). In comparison, those in the phase after diagnosis or who were in the process of receiving initial treatment had the least proportion of change (6.2%). Upon closer examination of those who reported changes in care and who were in the phase after initial treatment was completed, 72% reported a change in follow-up care, 20% a change in psycho-social counseling; yet, to a smaller extent, some reported changes to a planned operation, to systemic treatment, to radiotherapy, or to progress monitoring during treatment. This indicates that patients reporting changes in treatment may have been considering treatments that were planned months prior to completing the survey. The survey item asks if a change in treatment occurred during the pandemic; however, the exact date of reported change was not captured. It could be that the patient was in the phase after initial treatment when answering the questionnaire, but when responding to questions about change in treatment was considering an earlier time in the pandemic when she or he was in the initial phase, or the change may have occurred in the 4 months of the pandemic before the current study began. However, of the patients who took the survey during the first observation period (July 10-November 1, 2020), 17.6% reported a change in cancer care; 11.9% in the second period (November 2, 2020–March 11, 2021); and 9.3% in the third (March 12-June 30, 2021). This indicates that changes in care decreased over time.

With regard to the types of changes to cancer care and occurrence, our results are in line with those of a survey of German comprehensive cancer centers, starting at the end of March 2020, where most changes occurred earlier in the pandemic and mostly concerned follow-up appointments and counseling, while acute care was much less affected (reductions from 10 to 20%) (26). A survey of gynecological cancer patients in 16 European Union countries, conducted in May 2020, found that 36% had a change in their care plan, and 71% were concerned about cancer progression if there was a change in care (27). In a US survey of ovarian cancer patients, of the 43% who were in active treatment during the first 2 weeks of April 2020, 33% had a delay in some component of their cancer care, 26% specific to a planned surgery. Notably, delays in care were predictive of anxiety and depression (28). As these aforementioned studies occurred in the spring of 2020, during the initial phase of the pandemic, they cannot be directly compared to the results of our study, which began in July 2020. Partially overlapping with the timing of our study, the organization Cancer Australia reported a decrease in diagnostic and therapeutic services from March to May 2020 in contrast to the previous year; however, there was a partial recovery by June and a full recovery by September, excluding a few surgical procedures for breast, colorectal, and melanoma cancers (29).

### COVID-19 Related Concerns

Apart from changes to care, survey participants expressed worry about issues caused by the pandemic and its ensuing restrictions. Women were more likely to report worry regarding family and friends getting sick or dying, as well as missing contact with relatives and friends. Furthermore, concern about being infected was highest during the period of the second wave (November 2020 to March 2021) and lowest during the third wave (March to June 2021). This finding may be explained due to the first observation period (June to October 2020) occurring after the first initial higher infection rate had receded and restrictions on contact had been loosened. Regarding the third wave, although not addressed on the survey, COVID-19 vaccines were available during this observation period and presumably may have offered patients who were vaccinated some peace of mind. Interestingly, patients with statutory vs. private insurance were more worried that the pandemic could negatively impact the quality of their medical care. This corresponds to findings that privately insured patients have easier access to innovative medications, have shorter waiting times for appointments, and receive more time with physicians (30–32). Thus, some patients may view patient care with statutory health insurance as “worse” than that of private insurance. It's worth reiterating, we found no significant difference regarding change in care and insurance type (11% private insurance vs. 13% with statutory).

### Anxiety and Depression

More than half of survey participants (54.6%) reported symptoms for anxiety (HADS-A score ≥ 8) and 38.8% reported symptoms for depression (HADS-D score ≥ 8). Worry about possible negative effects of the pandemic on medical care was associated with anxiety symptoms. Of those who reported having symptoms of anxiety, 73.9% reported being concerned about the quality of their medical care vs. 45.2% who did not indicate concern over care. Aside from COVID-related factors, those who had attained an intermediate school-leaving certificate (leaving school after grade 10) were more likely to report symptoms of anxiety compared to those with a higher level of education (65 vs. 52% with a university entrance qualification). Interestingly, 45% of those with the lowest level of education (secondary general school-leaving certificate; leaving school after grade 9) reported symptoms of anxiety. Taken together, these findings make it difficult to draw conclusions about education level and anxiety for this study. For those reporting depressive symptoms (38.8% of study sample; HADS-D score ≥8), patients who had effective changes to care were more susceptible, but also those who reported worry about the quality of care. Patients who did not know if the cancer had metastasized were also more likely to report symptoms of depression.

Similar to our results, Frey et al. (28), found that 51.4% of their sample reported symptoms of anxiety and 26.5% symptoms of depression. Age (younger than 65) was predictive of greater cancer worry, anxiety, and depression and delay in cancer care was predictive of anxiety and depression. A survey conducted in the EU with gynecological cancer patients found that having experienced modifications of care due to the pandemic predictive of high depression scores (27). A study conducted in China also found that worry over cancer management due to COVID-19 was a predominant risk factor for psychological stress (33). Ayubi et al. conducted a meta-analysis of studies mostly occurring during the first 6 months of 2020, evaluating the level of depression and anxiety in cancer patients during the COVID-19 pandemic. Compared to control groups, cancer patients had higher anxiety levels. Studies using the HADS had an overall prevalence of 28% for depression (HADS-D ≥ 8), and a prevalence of 36% for anxiety (HADS-A ≥ 8) (22). Compared to before the onset of the pandemic, cancer patients had higher anxiety levels. Females and younger people reported higher mental burden (34). With regard to mental health in the general population in Germany during the pandemic, Bäuerle et al. (35) also found increased prevalence of anxiety (44.9%), depression (14.3%), psychological distress (65.2%) and COVID-19-related fear (59%).

### Limitations and Advantages

Our study had some limitations. The study began in July 2020, after the initial spike in incidence rates and after the first government imposed restrictions had been eased. Therefore, we were unable to capture changes in treatment as well as reactions to the pandemic writ large during and after the initial “lockdown” in March 2020. In addition we did not have baseline depression and anxiety measures pre-pandemic for our study sample. Thus, it is difficult to judge what symptoms are attributable to the pandemic and to what degree, aside from comparing data to other pre-pandemic studies. Another limitation of this study is the use of a non-population-based convenience sample.

Limitations in terms of the online administration via email should also be taken into consideration. While there is some concern over the ethics of recruitment via email (36, 37), the crux of the argument seems to stem over unwanted solicitation (i.e., spam). For the present study, the email invitation is sent as a response to an email initiated by the potential participants. The anonymity of the survey is explicitly stated in the invitation to the questionnaire and in the participant information. Further, data protection is explained in the participant information to address confidentiality.

Lastly, questions addressing the psychological distress unique to the pandemic were self-developed. These items were not developed to be aggregated into a single scale score. Therefore, there is no measure of internal consistency. Furthermore, we conducted cognitive pre-tests to qualitatively study and increase the validity of the items. However, various types of validity (such as construct validity) have not been assessed quantitatively. It might therefore be the case that some confounding factors that are not addressed by our survey influenced the responses to our self-developed items.

Despite some limitations, the current study provides detailed insight into the actual (i.e., changes in services) and perceived (e.g., psycho-social) burdens of a broad range of patients and disease conditions in real time over the course of 1 year. As of this writing, other publications examining the effects of the pandemic covered only the early phase. Moreover, in contrast to clinical research and face-to-face interviews, a “contact-free” online survey has obvious advantages during a pandemic. In addition, we had easy, yet controlled access to the group of interest. Our sample was well characterized, which allowed detailed analyses of subgroups.

## Conclusions and Future Directions

Our results indicate that the majority of cancer patients contacting the Cancer Information Service during the COVID-19 pandemic did not experience a change in primary cancer treatment. Of those with changes, appointments regarding follow-up care were more likely to be rescheduled or canceled. This indicates that most patients in our study received the initial medical treatment that they were scheduled to receive. However, the level of anxiety and psycho-social burden of cancer patients during the pandemic remained high, particularly for those patients who experienced a change in their care. Identifying risk factors of depression and anxiety among patients with cancer during the COVID-19 pandemic is crucial for taking adequate measures and for allocation of appropriate services. We found that patients who had a change in cancer care were more than twice as likely to have symptoms of depression as those with no changes. Studies suggest that psychological distress may lead to higher rates of mortality in cancer patients (38). This compounded with the effects of a potential delay in medical care (8) may decrease the odds of survivorship. Another factor predicting both anxiety and depression was worry that the pandemic would lessen the quality of their medical care. One survey respondent wrote in the comment section of the survey, “As a cancer patient, one feels relatively left alone after the end of the initial treatment. I am waiting 14 weeks for the follow-up treatment. There is only Corona, all other diseases are no longer important. There are many questions that no one answers…” Clear and open communication between doctors/oncologists and their patients might help assure patients that they will receive the proper care they need. While some delays in treatment and follow-up care may be to prevent the patient from exposure to the coronavirus, this should be expressly communicated to the patient.

Research on the COVID-19 pandemic's impact on both physical and mental health is important (and not lacking), but it is also crucial to continue research into non-COVID medical conditions to identify disparities, gaps, shortcomings and vulnerable groups so that health care management can address respective needs and ascertain equitable care to all patients. The provision of appropriate psychological support for those in need and the provision of transparency and comprehensible information are crucial. An implication of our study for health care services and policy makers is the need to assess and to attend to cancer patients' medical needs, with added emphasis on patients' psychological and social well-being. This applies particularly in situations where the system is strained and prioritization is necessary.

Future analyses will address in more detail the changes made to cancer care and examine the financial impact of the pandemic on cancer patients.

## Data Availability Statement

The raw data supporting the conclusions of this article will be made available by the authors, without undue reservation.

## Ethics Statement

The studies involving human participants were reviewed and approved by Ethics Committee University of Heidelberg. The patients/participants provided their written informed consent to participate in this study.

## Author Contributions

RE and AG: drafted the manuscript and literature review. EK: data processing. RE: data analysis. AG and JU: cognitive pretesting. All authors were involved in the conceptualization of this study, design of the questionnaire, and contributed to this manuscript.

## Conflict of Interest

The authors declare that the research was conducted in the absence of any commercial or financial relationships that could be construed as a potential conflict of interest.

## Publisher's Note

All claims expressed in this article are solely those of the authors and do not necessarily represent those of their affiliated organizations, or those of the publisher, the editors and the reviewers. Any product that may be evaluated in this article, or claim that may be made by its manufacturer, is not guaranteed or endorsed by the publisher.

## References

[1] Robert Koch Institut. Coronavirus Disease 2019 (COVID-19). Daily situation report of the Robert Koch Institute 30/04/2020 – Updated Status for Germany. (2020). Available online at: https://www.rki.de/DE/Content/InfAZ/N/Neuartiges_Coronavirus/Situationsberichte/2020-04-30-en.pdf?__blob=publicationFile (accessed August 27, 2021).

[2] Robert Koch Institut. Coronavirus Disease 2019 (COVID-19). Robert Koch-Institut: COVID-19-Dashboard (2021). Available online at: https://experience.arcgis.com/experience/478220a4c454480e823b17327b2bf1d4 (accessed September 23, 2021).

[3] Bundesministerium für Gesundheit. Coronavirus SARS-CoV-2: Chronik der bisherigen Maßnahmen. (2020). Available online at: https://www.bundesgesundheitsministerium.de/coronavirus/chronik-coronavirus.html (accessed April 30, 2020).

[4] Marron JM Joffe S Jagsi R Spence RA Hlubocky FJ. Ethics and resource scarcity: ASCO recommendations for the oncology community during the COVID-19 pandemic. J Clin Oncol. (2020) 38:2201–5. 10.1200/JCO.20.00960 32343643

[5] März JW Holm S Schlander M. Resource allocation in the Covid-19 health crisis: are Covid-19 preventive measures consistent with the Rule of Rescue? Med Health Care Philos. (2021) 24:487492. 10.1007/s11019-021-10045-0 34398349 PMC8364940

[6] Schlander M. Allen patienten gerecht werden: Gedanken eines Gesundheitsökonomen zur COVID-19-Krise. Frankfurter Allgemeine Zeitung. (2020). p. 20.

[7] Winkler E Maier-Hein L Baumann M. Kollaterale Depriorisierung - Zur Priorisierung überlebenswichtiger medizinischer Ressourcen. Die Politische Meinung. (2021). Available online at: https://www.kas.de/de/web/die-politische-meinung/artikel/detail/-/content/kollaterale-depriorisierung (accessed September 16, 2021).

[8] Hanna TP King WD Thibodeau S Jalink M Paulin GA Harvey-Jones E. Mortality due to cancer treatment delay: systematic review and meta-analysis. BMJ. (2020) 371:m4087. 10.1136/bmj.m4087 33148535 PMC7610021

[9] Wang Q Berger N Xu R. Analyses of risk, racial disparity, and outcomes among US patients with cancer and COVID-19 infection. JAMA Oncol. (2021) 7:220–7. 10.1001/jamaoncol.2020.6178 33300956 PMC7729584

[10] Parohan M Yaghoubi S Seraji A Javanbakht MH Sarraf P Djalali M. Risk factors for mortality in patients with Coronavirus disease 2019 (COVID-19) infection: a systematic review and meta-analysis of observational studies. Aging Male. (2020) 5:1416–24. 10.1080/13685538.2020.1774748 32508193

[11] Galea S Merchant RM Lurie N. The mental health consequences of COVID-19 and physical distancing: the need for prevention and early intervention. JAMA Intern Med. (2020) 180:6. 10.1001/jamainternmed.2020.1562 32275292

[12] Sojli E Tham WW Bryant R McAleer M. COVID-19 restrictions and age-specific mental health—US probability-based panel evidence. Transl Psychiatry. (2021) 11:418. 10.1038/s41398-021-01537-x 34349100 PMC8336527

[13] Pitman A Suleman S Hyde N Hodgkiss A. Depression and anxiety in patients with cancer. BMJ. (2018) 361:k1415. 10.1136/bmj.k1415 29695476

[14] Kuhnt S Brähler E Faller H Härter M Keller M Schulz H. Twelve-month and lifetime prevalence of mental disorders in cancer patients. Psychother Psychosom. (2016) 85:289–96. 10.1159/000446991 27508418

[15] Mehnert A Brähler E Faller H Härter M Keller M Schulz H. Four-week prevalence of mental disorders in patients with cancer across major tumor entities. J Clin Oncol. (2014) 32:3540–6. 10.1200/JCO.2014.56.0086 25287821

[16] Ashi N Kataoka Y Takemura T Shirakawa C Okazaki K Sakurai A. Factors influencing social isolation and loneliness among lung cancer patients: a cross-sectional study. Anticancer Res. (2020) 40:7141–5. 10.21873/anticanres.14744 33288614

[17] Rosset M Reifegerste D Baumann E Kludt E Weg-Remers S. Langzeittrends beim Krebsinformationsdienst (KID) des Deutschen Krebsforschungszentrums (DKFZ). Bundesgesundheitsblatt Gesundheitsforschung Gesundheitsschutz. (2019) 62:1120–8. 10.1007/s00103-019-02996-w 31410522

[18] Zigmond AS Snaith RP. The hospital anxiety and depression scale. Acta Psychiatr Scand. (1983) 67:6. 10.1111/j.1600-0447.1983.tb09716.x 6880820

[19] Herrmann C Buss U. Vorstellung und validierung einer deutschen version der “Hospital Anxiety And Depression Scale”(HAD-Skala). Ein Fragebogen zur Erfassung des psychischen Befindens bei Patienten mit körperlichen Beschwerden. Diagnostica. (1994) 40:143–54.

[20] Herrmann-Lingen C Buss U Snaith R. HADS-D: Hospitality Anxiety and Depression Scale: Deutsche Version: Ein Fragebogen zur Erfassung von Angst und Depressivität in der Somatischen Medizin Bern: Huber (2005).

[21] Bursac Z Gauss CH Williams DK Hosmer DW. Purposeful selection of variables in logistic regression. Source Code Biol Med. (2008) 16:17. 10.1186/1751-0473-3-17 19087314 PMC2633005

[22] Ayubi E Bashirian S Khazaei S. Depression and anxiety among patients with cancer during COVID-19 pandemic: a systematic review and meta-analysis. J Gastrointest Cancer. (2021) 52:499–507. 10.1007/s12029-021-00643-9 33950368 PMC8096890

[23] Statistisches Bundesamt (Destatis). Geschlecht, Altersgruppen, Allgemeine Schulausbildung. (2021). Available online at: https://www-genesis.destatis.de/genesis/online (accessed August, 28, 2021).

[24] Robert Koch Institut Zentrum für Krebsregisterdaten. Krebsarten. (2021). Available online at: https://www.krebsdaten.de/Krebs/DE/Home/homepage_node.html (accessed August 28, 2021).

[25] Statistisches Bundesamt (Destatis). Bevölkerung: Deutschland, Jahre (bis 2019), Geschlecht, Krankenkasse/Krankenversicherung. (2021). Available online at: https://www-genesis.destatis.de/genesis/online (accessed August 28, 2021).

[26] Fröhling S Arndt V. Versorgung von Krebspatienten: Corona-Effekt in der Onkologie. Dtsch Ärztebl. (2020) 117:A-2234/B-1893. Available online at: https://www.aerzteblatt.de/archiv/216717/Versorgung-von-Krebspatienten-Corona-Effekt-in-der-Onkologie

[27] Gultekin M Ak S Ayhan A Strojna A Pletnev A Fagotti A. Perspectives, fears and expectations of patients with gynaecological cancers during the COVID-19 pandemic: A Pan-European study of the European Network of Gynaecological Cancer Advocacy Groups (ENGAGe). Cancer Med. (2021) 10:208–19. 10.1002/cam4.3605 33205595 PMC7753798

[28] Frey MK Ellis AE Zeligs K Chapman-Davis E Thomas C Christos PJ. Impact of the coronavirus disease 2019 pandemic on the quality of life for women with ovarian cancer. Am J Obstet Gynecol. (2020) 223:725.e1–9. 10.1016/j.ajog.2020.06.049 32598911 PMC7318934

[29] Cancer Australia. National and Jurisdictional Data on the Impact of COVID-19 on Medical Services and Procedures in Australia: Breast, Colorectal, Lung, Prostate and Skin Cancers. Surry Hills, NSW: Cancer Australia (2020).

[30] Huber J Mielck A. Morbidität und Gesundheitsversorgung bei GKV-und PKV-Versicherten. Bundesgesundheitsblatt Gesundheitsforschung Gesundheitsschutz. (2010) 53:925–38. 10.1007/s00103-010-1119-7 20853090

[31] Lee S Gross SE Pfaff H Dresen A. Differences in perceived waiting time by health insurance type in the inpatient sector: an analysis of patients with breast cancer in Germany. Inquiry. (2019) 56:0046958019875897. 10.1177/0046958019875897 31526189 PMC6749783

[32] Ramos AL Hoffmann F Spreckelsen O. Waiting times in primary care depending on insurance scheme in Germany. BMC Health Serv Res. (2018) 18:191. 10.1186/s12913-018-3000-6 29558925 PMC5859448

[33] Wang Y Duan Z Ma Z Mao Y Li X Wilson A. Epidemiology of mental health problems among patients with cancer during COVID-19 pandemic. Transl Psychiatry. (2020) 10:263. 10.1038/s41398-020-00950-y 32737292 PMC7393344

[34] Bäuerle A Musche V Schmidt K Schweda A Fink M Weismüller B. Mental health burden of German cancer patients before and after the outbreak of COVID-19: predictors of mental health impairment. Int J Environ Res Public Health. (2021) 18:2318. 10.3390/ijerph18052318 33652949 PMC7967708

[35] Bäuerle A Teufel M Musche V Weismüller B Kohler H Hetkamp M. Increased generalized anxiety, depression and distress during the COVID-19 pandemic: a cross-sectional study in Germany. J Public Health. (2020) 42:672–8. 10.1093/pubmed/fdaa106 32657323 PMC7454766

[36] Krishnamurthy S. The ethics of conducting e-mail surveys. In Information Security and Ethics: Concepts, Methodologies, Tools, and Applications. Hershey, PA: IGI Global (2008). p. 3953–67. 10.4018/978-1-59904-937-3.ch268

[37] Sheehan KB Hoy MG. Using e-mail to survey Internet users in the United States: methodology and assessment. J Comput Mediat Commun. (1999) 4:JCMC435.

[38] Wang Y-H Li J-Q Shi J-F Que J-Y Liu J-J Lappin JM. Depression and anxiety in relation to cancer incidence and mortality: a systematic review and meta-analysis of cohort studies. Mol Psychiatry. (2020) 25:1487–99. 10.1038/s41380-019-0595-x 31745237

