# Supplementary material for: The COVID-19 Pandemic and Cancer Patients in Germany: Impact on Treatment, Follow-Up Care and Psychological Burden
Source: Front Public Health. 2022 Feb 9;9:788598. doi: 10.3389/fpubh.2021.788598 (PMC8865576; doi:10.3389/fpubh.2021.788598)
Supplement: Supplementary file 1 [file Data_Sheet_1.docx]

Supplementary Material

# Supplementary Tables

| **Table 1 \| Patients with changes in cancer care vs. those with no changes^a^** | | | | |  |
| --- | --- | --- | --- | --- | --- |
| **Total sample (*n*=621)** | | *n* (%) | *n* (%) | *X^2^* (df) | *P*-value |
|  |  | No change | Change |  |  |
| Age (*n*=586) | 18-40 | 30 (81.1) | 7 (18.9) | 2.62 (2) | 0.270 |
|  | 41-65 | 320 (86.5) | 50 (13.5) |  |  |
|  | 66+ | 161 (89.9) | 18 (10.1) |  |  |
| Gender (*n*=609) | Male | 130 (90.3) | 14 (9.7) | 1.61 (1) | 0.205 |
|  | Female | 401 (86.2) | 64 (13.8) |  |  |
| Education (*n*=600) | Secondary general school-leaving certificate | 55 (93.2) | 4 (6.8) | 2.27 (2) | 0.321 |
|  | Intermediate school-leaving certificate | 134 (85.9) | 22 (14.1) |  |  |
|  | University entrance qualification | 333 (86.5) | 52 (13.5) |  |  |
| Living situation | Lives alone | 94 (86.2) | 15 (13.8) | 0.11 (1) | 0.744 |
| (*n*=593) | Lives with others | 423 (87.4) | 61 (12.6) |  |  |
| Minor-aged kids at | No | 446 (88.0) | 61 (12.0) | 2.13 (1) | 0.144 |
| home (*n*=611) | Yes | 86 (82.7) | 18 (17.3) |  |  |
| Employment status | Employed | 230 (85.8) | 38 (14.2) | 6.22 (3) | 0.102 |
| (*n*=600) | Self-employed | 33 (80.5) | 8 (19.5) |  |  |
|  | Retired | 211 (90.6) | 22 (9.4) |  |  |
|  | Unemployed or not employed | 47 (81.0) | 11 (19.0) |  |  |
| Type of insurance | Private | 95 (88.8) | 12 (11.2) | 0.26 (2) | 0.877 |
| health insurance | Statutory | 320 (87.0) | 48 (13.0) |  |  |
| (*n*=581) | Statutory with private as supplement | 93 (87.7) | 13 (12.3) |  |  |
| Type of cancer | Breast cancer | 259 (84.6) | 47 (15.4) | 3.29 (2) | 0.193 |
| (*n*=609) | Prostate cancer | 58 (87.9) | 8 (12.1) |  |  |
|  | Other type | 213 (89.9) | 24 (10.1) |  |  |
| Metastatic cancer | No | 355 (87.2) | 52 (12.8) | 4.20 (2) | 0.122 |
| (*n*=605) | Yes or suspected | 137 (89.0) | 17 (11.0) |  |  |
|  | Do not know | 34 (77.3) | 10 (22.7) |  |  |
| Phase of treatment | After diagnosis or during initial treatment | 180 (93.8) | 12 (6.2) | 15.26 (3) | **0.002** |
| (*n*=585) | Initial treatment completed | 197 (81.1) | 46 (18.9) |  |  |
|  | Relapse/relapse treatment | 74 (88.1) | 10 (11.9) |  |  |
|  | Advanced disease/palliative treatment | 56 (84.8) | 10 (15.2) |  |  |
| Setting for main | Inpatient in hospital | 23 (79.3) | 6 (20.7) | 4.91 (3) | 0.178 |
| treatment (*n*=607) | Hospital outpatient | 160 (86.0) | 26 (14.0) |  |  |
|  | Oncology practice | 143 (91.7) | 13 (8.3) |  |  |
|  | Other | 203 (86.0) | 33 (14.0) |  |  |
| Sickened by | No or do not know | 517 (87.2) | 76 (12.8) | ^b^N/A | 0. 228 |
| COVID-19 (*n*=600) | Yes | 5 (71.4) | 2 (28.6) |  |  |
| Family, friend or | No or do not know | 421 (87.7) | 59 (12.3) | 0.81 (1) | 0.368 |
| acquaintance sickened | Yes | 111 (84.7) | 20 (15.3) |  |  |
| by COVID-19 (*n*=611) |  |  |  |  |  |
| ^a^Due to the COVID-19 pandemic, have you had a change to your treatment or aftercare plan?  ^b^Not Applicable. More than 20% of cells with expected count less than 5. Fisher’s Exact Test was conducted. | | | | | |

| **Table 2 \| Patient worry regarding being sickened or dying from COVID-19^a^** | | | | |  |
| --- | --- | --- | --- | --- | --- |
| **Total sample (*n*=621)** | | *n* (%) | *n* (%) | *χ^2^* (df) | *P*-value |
|  |  | Worried^b^ | Not Worried^c^ |  |  |
| Age (*n*=592) | 18-40 | 18 (46.2) | 21 (53.8) | 5.36 (2) | 0.069 |
|  | 41-65 | 110 (29.6) | 262 (70.4) |  |  |
|  | 66+ | 50 (27.6) | 131 (72.4) |  |  |
| Gender (*n*=616) | Male | 33 (22.8) | 112 (77.2) | 4.58 (1) | **0.032** |
|  | Female | 151 (32.1) | 320 (67.9) |  |  |
| Education (*n*=607) | Secondary general school-leaving certificate | 19 (31.1) | 42 (68.9) | 1.33 (2) | 0.514 |
|  | Intermediate school-leaving certificate | 53 (33.5) | 105 (66.5) |  |  |
|  | University entrance qualification | 111 (28.6) | 277 (71.4) |  |  |
| Living situation | Lives alone | 38 (34.2) | 73 (65.8) | 1.07 (1) | 0.301 |
| (*n*=600) | Lives with others | 143 (29.2) | 346 (70.8) |  |  |
| Minor-aged kids at | No | 149 (29.1) | 363 (70.9) | 1.00 (1) | 0.320 |
| home (*n*=618) | Yes | 36 (34.0) | 70 (66.0) |  |  |
| Employment status | Employed | 85 (31.5) | 185 (68.5) | 1.62 (3) | 0.665 |
| (*n*=607) | Self-employed | 13 (32.5) | 27 (67.5) |  |  |
|  | Retired | 69 (29.0) | 169 (71.0) |  |  |
|  | Unemployed or not employed | 14 (23.7) | 45 (76.3) |  |  |
| Type of insurance | Private | 25 (23.6) | 81 (76.4) | 2.57 (2) | 0.442 |
| health insurance | Statutory | 119 (31.6) | 257 (68.4) |  |  |
| (*n*=588) | Statutory with private as supplement | 32 (30.2) | 74 (69.8) |  |  |
| Type of cancer | Breast cancer | 102 (32.9) | 208 (67.1) | 10.15 (4) | **0.038** |
| (*n*=616) | Prostate cancer | 10 (15.4) | 55 (84.6) |  |  |
|  | Colon cancer | 8 (36.4) | 14 (63.6) |  |  |
|  | Lung cancer | 3 (15.8) | 16 (84.2) |  |  |
|  | Other | 61 (30.5) | 139 (69.5) |  |  |
| Metastatic cancer | No | 355 (87.2) | 52 (12.8) | 2.81 (2) | 0.245 |
| (*n*=605) | Yes or suspected | 137 (89.0) | 17 (11.0) |  |  |
|  | Do not know | 34 (77.3) | 10 (22.7) |  |  |
| Phase of treatment | After diagnosis or during initial treatment | 58 (29.6) | 138 (70.4) | 0.27 (3) | 0.965 |
| (*n*=592) | Initial treatment completed | 75 (30.7) | 169 (69.3) |  |  |
|  | Relapse/relapse treatment | 27 (31.8) | 58 (68.2) |  |  |
|  | Advanced disease/palliative treatment | 19 (28.4) | 48 (71.6) |  |  |
| Setting for main | Inpatient in hospital | 8 (25.8) | 23 (74.2) | 0.55 (3) | 0.908 |
| treatment (*n*=614) | Hospital outpatient | 54 (29.0) | 132 (71.0) |  |  |
|  | Oncology practice | 50 (31.6) | 108 (68.4) |  |  |
|  | Other | 72 (30.1) | 167 (69.9) |  |  |
| Observation period | Before second wave (Jul. 10 – Nov. 1, 2020) | 56 (30.4) | 128 (69.6) | 13.10 (2) | **0.001** |
| (*n*=618) | Second wave (Nov. 2, 2020 – Mar. 11, 2021) | 100 (35.6) | 181 (64.4) |  |  |
|  | Third wave (Mar. 12, 2021 – Jun. 30, 2021) | 29 (19.0) | 124 (81.0) |  |  |
| Self sickened by | No or do not know | 181 (30.5) | 412 (69.5) | N/A^d^ | 0.681 |
| COVID-19 (*n*=600) | Yes | 1 (14.3) | 6 (85.7) |  |  |
| Family, friend or | No or do not know | 143 (29.9) | 336 (70.1) | 0.01 (1) | 0.935 |
| acquaintance | Yes | 42 (30.2) | 97 (69.8) |  |  |
| sickened by |  |  |  |  |  |
| COVID-19 (*n*=618) |  |  |  |  |  |
| ^a^I am worried that I could be sickened or die from the coronavirus infection. ^b^Agree and strongly agree ^c^Neutral to strongly disagree  ^d^Not Applicable. More than 20% of cells with expected count less than 5. Fisher’s Exact Test was conducted. | | | | | |

| **Table 3 \| Patient worry regarding relatives and friends being sickened or dying from COVID-19^a^** | | | | |  |
| --- | --- | --- | --- | --- | --- |
| **Total sample (*n*=621)** | | *n* (%) | *n* (%) | *χ^2^* (df) | *P*-value |
|  |  | Worried^b^ | Not Worried^c^ |  |  |
| Age (*n*=591) | 18-40 | 17 (43.6) | 22 (56.4) | 0.56 (2) | 0.755 |
|  | 41-65 | 161 (43.3) | 211 (56.7) |  |  |
|  | 66+ | 72 (40.0) | 108 (60.0) |  |  |
| Gender (*n*=615) | Male | 46 (31.9) | 98 (68.1) | 7.73 (1) | **0.005** |
|  | Female | 212 (45.0) | 259 (55.0) |  |  |
| Education (*n*=606) | Secondary general school-leaving certificate | 28 (45.9) | 33 (54.1) | 1.41 (2) | 0.494 |
|  | Intermediate school-leaving certificate | 71 (45.2) | 86 (54.8) |  |  |
|  | University entrance qualification | 157 (40.5) | 231 (59.5) |  |  |
| Living situation | Lives alone | 50 (45.0) | 61 (55.0) | 0.44 (1) | 0.507 |
| (*n*=599) | Lives with others | 203 (41.6) | 285 (58.4) |  |  |
| Minor-aged kids at | No | 211 (41.3) | 300 (58.7) | 0.88 (1) | 0.349 |
| home (*n*=617) | Yes | 49 (46.2) | 57 (53.8) |  |  |
| Employment status | Employed | 116 (43.0) | 154 (57.0) | 0.64 (3) | 0.887 |
| (*n*=606) | Self-employed | 19 (47.5) | 21 (52.5) |  |  |
|  | Retired | 98 (41.4) | 139 (58.6) |  |  |
|  | Unemployed or not employed | 24 (40.7) | 35 (59.3) |  |  |
| Type of insurance | Private | 37 (34.9) | 69 (65.1) | 3.07 (2) | 0.215 |
| health insurance | Statutory | 166 (44.3) | 209 (55.7) |  |  |
| (*n*=587) | Statutory with private as supplement | 43 (40.6) | 63 (59.4) |  |  |
| Type of cancer | Breast cancer | 143 (46.1) | 167 (53.9) | 6.32 (4) | 0.177 |
| (*n*=615) | Prostate cancer | 19 (29.7) | 45 (70.3) |  |  |
|  | Colon cancer | 9 (40.9) | 13 (59.1) |  |  |
|  | Lung cancer | 8 (42.1) | 11 (57.9) |  |  |
|  | Other type | 81 (40.5) | 119 (59.5) |  |  |
| Metastatic cancer | No | 186 (45.3) | 225 (54.7) | 5.59 (2) | 0.062 |
| (*n*=611) | Yes or suspected | 57 (36.5) | 99 (63.5) |  |  |
|  | Do not know | 14 (31.8) | 30 (68.2) |  |  |
| Phase of treatment | After diagnosis or during initial treatment | 70 (35.7) | 126 (64.3) | 7.48 (3) | 0.058 |
| (*n*=591) | Initial treatment completed | 118 (48.6) | 125 (51.4) |  |  |
|  | Relapse/relapse treatment | 36 (42.4) | 49 (57.6) |  |  |
|  | Advanced disease/palliative treatment | 27 (40.3) | 40 (59.7) |  |  |
| Setting for main | Inpatient in hospital | 12 (38.7) | 19 (61.3) | 1.83 (3) | 0.610 |
| treatment (*n*=613) | Hospital outpatient | 72 (38.7) | 114 (61.3) |  |  |
|  | Oncology practice | 67 (42.4) | 91 (57.6) |  |  |
|  | Other | 107 (45.0) | 131 (55.0) |  |  |
| Observation period | Before second wave (Jul. 10 – Nov. 1, 2020) | 69 (37.5) | 115 (62.5) | 14.40 (2) | **0.001** |
| (*n*=617) | Second wave (Nov. 2, 2020 – Mar. 11, 2021) | 141 (50.2) | 140 (49.8) |  |  |
|  | Third wave (Mar. 12, 2021 – Jun. 30, 2021) | 50 (32.9) | 102 (67.1) |  |  |
| Self sickened by | No or do not know | 251 (42.4) | 341 (57.6) | N/A^d^ | 0.143 |
| COVID-19 (*n*=599) | Yes | 5 (71.4) | 2 (28.6) |  |  |
| Family, friend or | No or do not know | 190 (39.7) | 288 (60.3) | 4.97 (1) | **0.026** |
| acquaintance | Yes | 70 (50.4) | 69 (49.6) |  |  |
| sickened by |  |  |  |  |  |
| COVID-19 (*n*=617) |  |  |  |  |  |
| ^a^I am worried that family or friends could be sickened or die from the coronavirus infection. ^b^Agree and strongly agree ^c^Neutral to strongly disagree  ^d^Not Applicable. More than 20% of cells with expected count less than 5. Fisher’s Exact Test was conducted. | | | | | |

| **Table 4 \| Patient worry regarding effects of the pandemic affecting the quality of medical treatment**^a^ | | | | |  |
| --- | --- | --- | --- | --- | --- |
| **Total sample (*n*=621)** | | *n* (%) | *n* (%) | *χ^2^* (df) | *P*-value |
|  |  | Worried^b^ | Not Worried^c^ |  |  |
| Age (*n*=591) | 18-40 | 20 (51.3) | 19 (48.7) | 8.69 (2) | **0.013** |
|  | 41-65 | 125 (33.6) | 247 (66.4) |  |  |
|  | 66+ | 49 (27.2) | 131 (72.8) |  |  |
| Gender (*n*=615) | Male | 47 (32.4) | 98 (67.6) | 0.30 (1) | 0.862 |
|  | Female | 156 (33.2) | 314 (66.8) |  |  |
| Education (*n*=606) | Secondary general school-leaving certificate | 22 (36.1) | 39 (63.9) | 0.52 (2) | 0.770 |
|  | Intermediate school-leaving certificate | 49 (31.2) | 108 (68.8) |  |  |
|  | University entrance qualification | 130 (33.5) | 258 (66.5) |  |  |
| Living situation | Lives alone | 41 (37.3) | 69 (62.7) | 1.00 (1) | 0.318 |
| (*n*=599) | Lives with others | 158 (32.3) | 331 (67.7) |  |  |
| Minor-aged kids at | No | 159 (31.1) | 352 (68.9) | 4.30 (1) | **0.038** |
| home (*n*=617) | Yes | 44 (41.5) | 62 (58.5) |  |  |
| Employment status | Employed | 98 (36.3) | 172 (63.7) | 5.04 (3) | 0.169 |
| (*n*=606) | Self-employed | 15 (37.5) | 25 (62.5) |  |  |
|  | Retired | 65 (27.4) | 172 (72.6) |  |  |
|  | Unemployed or not employed | 20 (33.9) | 39 (66.1) |  |  |
| Type of insurance | Private | 20 (19.0) | 85 (81.0) | 14.63 (2) | **0.001** |
| health insurance | Statutory | 143 (38.0) | 233 (62.0) |  |  |
| (*n*=587) | Statutory with private as supplement | 30 (28.3) | 76 (71.7) |  |  |
| Type of cancer | Breast cancer | 101 (32.6) | 209 (67.4) | 6.84 (4) | 0.145 |
| (*n*=615) | Prostate cancer | 16 (24.6) | 49 (75.4) |  |  |
|  | Colon cancer | 11 (50.0) | 11 (50.0) |  |  |
|  | Lung cancer | 4 (21.1) | 15 (78.9) |  |  |
|  | Other type | 71 (35.7) | 128 (64.3) |  |  |
| Metastatic cancer | No | 131 (31.8) | 281 (68.2) | 1.61 (2) | 0.447 |
| (*n*=611) | Yes or suspected | 53 (34.2) | 102 (65.8) |  |  |
|  | Do not know | 18 (40.9) | 26 (59.1) |  |  |
| Phase of treatment | After diagnosis or during initial treatment | 66 (33.7) | 130 (66.3) | 5.40 (3) | 0.145 |
| (*n*=591) | Initial treatment completed | 77 (31.6) | 167 (68.4) |  |  |
|  | Relapse/relapse treatment | 37 (43.5) | 48 (56.5) |  |  |
|  | Advanced disease/palliative treatment | 18 (27.3) | 48 (72.7) |  |  |
| Setting for main | Inpatient in hospital | 15 (48.4) | 16 (51.6) | 4.56 (3) | 0.207 |
| treatment (*n*=613) | Hospital outpatient | 57 (30.6) | 129 (69.4) |  |  |
|  | Oncology practice | 56 (35.7) | 101 (64.3) |  |  |
|  | Other | 75 (31.4) | 164 (68.6) |  |  |
| Observation period | Before second wave (Jul. 10 – Nov. 1, 2020) | 68 (37.0) | 116 (63.0) | 3.44 (2) | 0.179 |
| (*n*=617) | Second wave (Nov. 2, 2020 – Mar. 11, 2021) | 93 (33.2) | 187 (66.8) |  |  |
|  | Third wave (Mar. 12, 2021 – Jun. 30, 2021) | 42 (27.5) | 111 (72.5) |  |  |
| Self sickened by | No or do not know | 193 (32.6) | 399 (67.4) | N/A^d^ | 0.169 |
| COVID-19 (*n*=599) | Yes | 4 (57.1) | 3 (42.9) |  |  |
| Family, friend or | No or do not know | 151 (31.6) | 327 (68.4) | 1.65 (1) | 0.199 |
| acquaintance | Yes | 52 (37.4) | 87 (62.6) |  |  |
| sickened by |  |  |  |  |  |
| COVID-19 (*n*=617) |  |  |  |  |  |
| ^a^I am worried that the effects of the pandemic could affect the quality of medical treatment. ^b^Agree and strongly agree ^c^Neutral to strongly disagree  ^d^Not Applicable. More than 20% of cells with expected count less than 5. Fisher’s Exact Test was conducted. | | | | | |

| **Table 5 \| Patient missing contacts with relatives, friends, work colleagues, neighbors**^a^ | | | | |  |
| --- | --- | --- | --- | --- | --- |
| **Total sample (*n*=621)** | | *n* (%) | *n* (%) | *χ^2^* (df) | *P*-value |
|  |  | Missing Contact^b^ | Not Missing Contact^c^ |  |  |
| Age (*n*=594) | 18-40 | 31 (79.5) | 8 (20.5) | 5.38 (2) | 0.068 |
|  | 41-65 | 272 (72.9) | 101 (27.1) |  |  |
|  | 66+ | 118 (64.8) | 64 (35.2) |  |  |
| Gender (*n*=618) | Male | 80 (54.4) | 67 (45.6) | 24.15 (1) | **<0.001** |
|  | Female | 356 (75.6) | 115 (24.4) |  |  |
| Education (*n*=609) | Secondary general school-leaving certificate | 42 (67.7) | 20 (32.3) | 1.53 (2) | 0.464 |
|  | Intermediate school-leaving certificate | 118 (74.2) | 41 (25.8) |  |  |
|  | University entrance qualification | 269 (69.3) | 119 (30.7) |  |  |
| Living situation | Lives alone | 79 (71.2) | 32 (28.8) | 0.01 (1) | 0.917 |
| (*n*=602) | Lives with others | 347 (70.7) | 144 (29.3) |  |  |
| Minor-aged kids at | No | 358 (69.8) | 155 (30.2) | 1.06 (1) | 0.303 |
| home (*n*=620) | Yes | 80 (74.8) | 27 (25.2) |  |  |
| Employment status | Employed | 204 (75.3) | 67 (24.7) | 6.31 (3) | 0.097 |
| (*n*=609) | Self-employed | 29 (72.5) | 11 (27.5) |  |  |
|  | Retired | 158 (66.1) | 81 (33.9) |  |  |
|  | Unemployed or not employed | 38 (64.4) | 21 (35.6) |  |  |
| Type of insurance | Private | 68 (63.6) | 39 (36.4) | 3.81 (2) | 0.149 |
| health insurance | Statutory | 268 (71.1) | 109 (28.9) |  |  |
| (*n*=590) | Statutory with private as supplement | 80 (75.5) | 26 (24.5) |  |  |
| Type of cancer | Breast cancer | 235 (75.8) | 75 (24.2) | 26.04 (7) | **<0.001** |
| (*n*=618) | Prostate cancer | 30 (45.5) | 36 (54.5) |  |  |
|  | Colon cancer | 16 (72.7) | 6 (27.3) |  |  |
|  | Lung cancer | 16 (84.2) | 3 (15.8) |  |  |
|  | Other type | 140 (70.7) | 61 (29.3) |  |  |
| Metastatic cancer | No | 307 (74.2) | 107 (25.8) | 8.60 (2) | 0.14 |
| (*n*=614) | Yes or suspected | 102 (65.4) | 54 (34.6) |  |  |
|  | Do not know | 25 (56.8) | 19 (43.2) |  |  |
| Phase of treatment | After diagnosis or during initial treatment | 142 (72.1) | 55 (27.9) | 2.61 (3) | 0.456 |
| (*n*=594) | Initial treatment completed | 179 (73.1) | 66 (26.9) |  |  |
|  | Relapse/relapse treatment | 56 (65.9) | 29 (34.1) |  |  |
|  | Advanced disease/palliative treatment | 44 (65.7) | 23 (34.3) |  |  |
| Setting for main | Inpatient in hospital | 21 (67.7) | 10 (32.3) | 0.15 (3) | 0.985 |
| treatment (*n*=616) | Hospital outpatient | 133 (71.1) | 54 (28.9) |  |  |
|  | Oncology practice | 112 (70.4) | 47 (29.6) |  |  |
|  | Other | 169 (70.7) | 70 (29.3) |  |  |
| Observation period | Before second wave (Jul. 10 – Nov. 1, 2020) | 105 (56.8) | 80 (43.2) | 24.59 (2) | **<0.001** |
| (*n*=620) | Second wave (Nov. 2, 2020 – Mar. 11, 2021) | 217 (77.0) | 65 (23.0) |  |  |
|  | Third wave (Mar. 12, 2021 – Jun. 30, 2021) | 116 (75.8) | 37 (24.2) |  |  |
| Self sickened by | No or do not know | 421 (70.8) | 174 (29.2) | N/A^d^ | 0.204 |
| COVID-19 (*n*=602) | Yes | 3 (42.9) | 4 (57.1) |  |  |
| Family, friend or | No or do not know | 327 (68.0) | 154 (32.0) | 7.33 (1) | **0.007** |
| acquaintance | Yes | 111 (79.9) | 28 (20.1) |  |  |
| sickened by |  |  |  |  |  |
| COVID-19 (*n*=620) |  |  |  |  |  |
| ^a^How do you feel about the limitations of personal encounters due to the Corona pandemic? Personal contacts with relatives, friends, work colleagues, neighbors… ^b^Miss it extremely and miss it quite a bit ^c^Neutral, do not miss it a lot, do not miss it at all  ^d^Not Applicable. More than 20% of cells with expected count less than 5. Fisher’s Exact Test was conducted. | | | | | |

| **Table 6 \| Symptoms of anxiety^a^** | | | | |  |
| --- | --- | --- | --- | --- | --- |
| **Total sample (*n*=621)** | | *n* (%) | *n* (%) | *χ^2^* (df) | *P*-value |
|  |  | Yes | No |  |  |
| Age (*n*=595) | 18-40 | 26 (66.7) | 13 (33.3) | 11.59 (2) | **0.003** |
|  | 41-65 | 218 (58.4) | 155 (41.6) |  |  |
|  | 66+ | 82 (44.8) | 101 (55.2) |  |  |
| Gender (*n*=619) | Male | 64 (43.5) | 83 (56.5) | 9.52 (1) | **0.002** |
|  | Female | 274 (58.1) | 198 (41.9) |  |  |
| Education (*n*=610) | Secondary general school-leaving certificate | 28 (45.2) | 34 (54.8) | 10.00 (2) | **0.007** |
|  | Intermediate school-leaving certificate | 103 (64.8) | 56 (35.2) |  |  |
|  | University entrance qualification | 202 (51.9) | 187 (48.1) |  |  |
| Living situation | Lives alone | 67 (60.4) | 44 (39.6) | 1.74 (1) | 0.187 |
| (*n*=603) | Lives with others | 263 (53.5) | 229 (46.5) |  |  |
| Minor-aged kids at | No | 267 (51.9) | 247 (48.1) | 8.41 (1) | **0.004** |
| home (*n*=621) | Yes | 72 (67.3) | 35 (32.7) |  |  |
| Employment status | Employed | 163 (60.1) | 108 (39.9) | 11.77 (3) | **0.008** |
| (*n*=610) | Self-employed | 26 (63.4) | 15 (36.6) |  |  |
|  | Retired | 110 (46.0) | 129 (54.0) |  |  |
|  | Unemployed or not employed | 33 (55.9) | 26 (44.1) |  |  |
| Type of insurance | Private | 54 (50.0) | 54 (50.0) | 2.20 (2) | 0.333 |
| health insurance | Statutory | 214 (56.8) | 163 (43.2) |  |  |
| (*n*=591) | Statutory with private as supplement | 54 (50.9) | 52 (49.1) |  |  |
| Type of cancer | Breast cancer | 181 (58.4) | 129 (41.6) | 12.72 (4) | **0.013** |
| (*n*=619) | Prostate cancer | 23 (34.8) | 43 (65.2) |  |  |
|  | Colon cancer | 11 (47.8) | 12 (52.2) |  |  |
|  | Lung cancer | 10 (52.6) | 9 (47.4) |  |  |
|  | Other type | 112 (55.7) | 89 (44.3) |  |  |
| Metastatic cancer | No | 231 (55.8) | 183 (44.2) | 9.60 (2) | **0.008** |
| (*n*=615) | Yes or suspected | 74 (47.1) | 83 (52.9) |  |  |
|  | Do not know | 32 (72.7) | 12 (27.3) |  |  |
| Phase of treatment | After diagnosis or during initial treatment | 111 (56.3) | 86 (43.7) | 0.61 (3) | 0.894 |
| (*n*=595) | Initial treatment completed | 135 (55.1) | 110 (44.9) |  |  |
|  | Relapse/relapse treatment | 44 (51.8) | 41 (48.2) |  |  |
|  | Advanced disease/palliative treatment | 36 (52.9) | 32 (47.1) |  |  |
| Setting for main | Inpatient in hospital | 19 (61.3) | 12 (38.7) | 4.00 (3) | 0.265 |
| treatment (*n*=617) | Hospital outpatient | 111 (59.4) | 76 (40.6) |  |  |
|  | Oncology practice | 80 (50.0) | 80 (50.0) |  |  |
|  | Other | 126 (52.7) | 113 (47.3) |  |  |
| Change in cancer | No | 273 (51.3) | 259 (48.7) | 18.62 (1) | **<0.001** |
| care (*n*=611) | Yes | 61 (77.2) | 18 (22.8) |  |  |
| Worry about corona | No | 208 (48.0) | 225 (52.0) | 24.60 (1) | **<0.001** |
| infection (*n*=618) | Yes | 129 (69.7) | 56 (30.3) |  |  |
| Worry about family/ | No | 167 (46.8) | 190 (53.2) | 21.01 (1) | **<0.001** |
| friends corona | Yes | 170 (65.4) | 90 (34.6) |  |  |
| infection (*n*=617) |  |  |  |  |  |
| Worry about possible | No | 187 (45.2) | 227 (54.8) | 45.37 (1) | **<0.001** |
| effects on quality of | Yes | 150 (73.9) | 53 (26.1) |  |  |
| medical care (*n*=617) |  |  |  |  |  |
| Missing contact with | No | 83 (45.6) | 99 (54.4) | 8.25 (1) | **0.004** |
| relatives, friends, etc. | Yes | 255 (58.2) | 183 (41.8) |  |  |
| (*n*=620) |  |  |  |  |  |
| Observation period | Before second wave (Jul. 10 – Nov. 1, 2020) | 110 (59.5) | 75 (40.5) | 3.40 (2) | 0.183 |
| (*n*=621) | Second wave (Nov. 2, 2020 – Mar. 11, 2021) | 144 (50.9) | 139 (49.1) |  |  |
|  | Third wave (Mar. 12, 2021 – Jun. 30, 2021) | 85 (55.6) | 68 (44.4) |  |  |
| Self sickened by | No or do not know | 326 (54.7) | 270 (45.3) | N/A^b^ | 1.000 |
| COVID-19 (*n*=603) | Yes | 3 (42.9) | 4 (57.1) |  |  |
| Family, friend or | No or do not know | 262 (54.4) | 220 (45.6) | 0.05 (1) | 0.828 |
| acquaintance | Yes | 77 (55.4) | 62 (44.6) |  |  |
| sickened by |  |  |  |  |  |
| COVID-19 (*n*=621) |  |  |  |  |  |
| ^a^Hospital Anxiety and Depression Scale-Anxiety, anxiety score ≥ 8  ^b^Not Applicable. More than 20% of cells with expected count less than 5. Fisher’s Exact Test was conducted. | | | | | |

| **Table 7 \| Symptoms of depression^a^** | | | | |  |
| --- | --- | --- | --- | --- | --- |
| **Total sample (*n*=621)** | | *n* (%) | *n* (%) | *χ^2^* (df) | *P*-value |
|  |  | Yes | No |  |  |
| Age (*n*=595) | 18-40 | 14 (35.9) | 25 (64.1) | 1.26 (2) | 0.532 |
|  | 41-65 | 150 (40.2) | 223 (59.8) |  |  |
|  | 66+ | 65 (35.5) | 118 (64.5) |  |  |
| Gender (*n*=619) | Male | 54 (36.7) | 93 (63.3) | 0.39 (1) | 0.531 |
|  | Female | 187 (39.6) | 285 (60.4) |  |  |
| Education (*n*=610) | Secondary general school-leaving certificate | 29 (46.8) | 33 (53.2) | 8.77 (2) | 0.012 |
|  | Intermediate school-leaving certificate | 74 (46.5) | 85 (53.5) |  |  |
|  | University entrance qualification | 134 (34.4) | 255 (65.6) |  |  |
| Living situation | Lives alone | 51 (45.9) | 60 (54.1) | 3.06 (1) | 0.080 |
| (*n*=603) | Lives with others | 182 (37.0) | 310 (63.0) |  |  |
| Minor-aged kids at | No | 194 (37.7) | 320 (62.3) | 1.42 (1) | 0.233 |
| home (*n*=621) | Yes | 47 (43.9) | 60 (56.1) |  |  |
| Employment status | Employed | 103 (38.0) | 168 (62.0) | 4.16 (3) | 0.245 |
| (*n*=610) | Self-employed | 15 (36.6) | 26 (63.4) |  |  |
|  | Retired | 88 (36.8) | 151 (63.2) |  |  |
|  | Unemployed or not employed | 30 (50.8) | 29 (49.2) |  |  |
| Type of insurance | Private | 37 (34.3) | 71 (65.7) | 3.75 (2) | 0.153 |
| health insurance | Statutory | 155 (41.1) | 222 (58.9) |  |  |
| (*n*=591) | Statutory with private as supplement | 34 (32.1) | 72 (67.9) |  |  |
| Type of cancer | Breast cancer | 122 (39.4) | 188 (60.6) | 7.00 (4) | 0.136 |
| (*n*=619) | Prostate cancer | 16 (24.2) | 50 (75.8) |  |  |
|  | Colon cancer | 10 (43.5) | 13 (56.5) |  |  |
|  | Lung cancer | 8 (42.1) | 11 (57.9) |  |  |
|  | Other type | 84 (41.8) | 117 (58.2) |  |  |
| Metastatic cancer | No | 152 (36.7) | 262 (63.3) | 12.28 (2) | **0.002** |
| (*n*=615) | Yes or suspected | 59 (37.6) | 98 (62.4) |  |  |
|  | Do not know | 28 (63.6) | 16 (36.4) |  |  |
| Phase of treatment | After diagnosis or during initial treatment | 81 (41.1) | 116 (58.9) | 2.81 (3) | 0.422 |
| (*n*=595) | Initial treatment completed | 85 (34.7) | 160 (65.3) |  |  |
|  | Relapse/relapse treatment | 35 (41.2) | 50 (58.8) |  |  |
|  | Advanced disease/palliative treatment | 29 (42.6) | 39 (57.4) |  |  |
| Setting for main | Inpatient in hospital | 15 (48.4) | 16 (51.6) | 10.32 (3) | 0.16 |
| treatment (*n*=617) | Hospital outpatient | 88 (47.1) | 99 (52.9) |  |  |
|  | Oncology practice | 58 (36.2) | 102 (63.7) |  |  |
|  | Other | 79 (33.1) | 160 (66.9) |  |  |
| Change in cancer | No | 188 (35.3) | 344 (64.7) | 22.60 (1) | **<0.001** |
| care (*n*=611) | Yes | 50 (63.3) | 29 (36.7) |  |  |
| Worry about corona | No | 148 (34.2) | 285 (65.8) | 12.31 (1) | **<0.001** |
| infection (*n*=618) | Yes | 91 (49.2) | 94 (50.8) |  |  |
| Worry about family/ | No | 111 (31.1) | 246 (68.9) | 20.87 (1) | **<0.001** |
| friends corona | Yes | 128 (49.2) | 132 (50.8) |  |  |
| infection (*n*=617) |  |  |  |  |  |
| Worry about possible | No | 124 (30.0) | 290 (70.0) | 40.91 (1) | **<0.001** |
| effects on quality of | Yes | 115 (56.7) | 88 (43.3) |  |  |
| medical care (*n*=617) |  |  |  |  |  |
| Missing contact with | No | 69 (37.9) | 113 (62.1) | 0.07 (1) | 0.793 |
| relatives, friends, etc. | Yes | 171 (39.0) | 267 (61.0) |  |  |
| (*n*=620) |  |  |  |  |  |
| Observation period | Before second wave (Jul. 10 – Nov. 1, 2020) | 74 (40.0) | 111 (60.0) | 0.24 (2) | 0.887 |
| (*n*=621) | Second wave (Nov. 2, 2020 – Mar. 11, 2021) | 107 (37.8) | 176 (62.2) |  |  |
|  | Third wave (Mar. 12, 2021 – Jun. 30, 2021) | 60 (39.2) | 93 (60.8) |  |  |
| Self sickened by | No or do not know | 234 (39.3) | 362 (60.7) | N/A^b^ | 0.710 |
| COVID-19 (*n*=603) | Yes | 2 (28.6) | 5 (71.4) |  |  |
| Family, friend or | No or do not know | 192 (39.8) | 290 (60.2) | 0.95 (1) | 0.329 |
| acquaintance | Yes | 49 (35.3) | 90 (64.7) |  |  |
| sickened by |  |  |  |  |  |
| COVID-19 (*n*=621) |  |  |  |  |  |
| ^a^Hospital Anxiety and Depression Scale-Depression, depression score ≥ 8  ^b^Not Applicable. More than 20% of cells with expected count less than 5. Fisher’s Exact Test was conducted. | | | | | |
